# Recent Progress on Photoelectrochemical Water Splitting of Graphitic Carbon Nitride (g−CN) Electrodes

**DOI:** 10.3390/nano12142374

**Published:** 2022-07-11

**Authors:** Ying Zhu, Liang He, Yiqiang Ni, Genzhuang Li, Dongshuai Li, Wang Lin, Qiliang Wang, Liuan Li, Haibin Yang

**Affiliations:** 1 State Key Laboratory of Superhard Material, College of Physics, Jilin University, Changchun 130012, China; zhuying21@mails.jlu.edu.cn (Y.Z.); ligz20@mails.jlu.edu.cn (G.L.); lids21@mails.jlu.edu.cn (D.L.); linw20@mails.jlu.edu.cn (W.L.); yanghb@jlu.edu.cn (H.Y.); 2 No. 5 Electronics Research Institute of the Ministry of Industry and Information Technology, Guangzhou 510610, China; heliang@ceprei.com (L.H.); niyiqiang@ceprei.com (Y.N.); 3 Yibin Research Institute, Jilin University, Yibin 644000, China

**Keywords:** graphitic carbon nitride, photoelectrochemical, water splitting

## Abstract

Graphitic carbon nitride (g−CN), a promising visible-light-responsive semiconductor material, is regarded as a fascinating photocatalyst and heterogeneous catalyst for various reactions due to its non-toxicity, high thermal durability and chemical durability, and “earth-abundant” nature. However, practical applications of g−CN in photoelectrochemical (PEC) and photoelectronic devices are still in the early stages of development due to the difficulties in fabricating high-quality g−CN layers on substrates, wide band gaps, high charge-recombination rates, and low electronic conductivity. Various fabrication and modification strategies of g−CN-based films have been reported. This review summarizes the latest progress related to the growth and modification of high-quality g−CN-based films. Furthermore, (1) the classification of synthetic pathways for the preparation of g−CN films, (2) functionalization of g−CN films at an atomic level (elemental doping) and molecular level (copolymerization), (3) modification of g−CN films with a co-catalyst, and (4) composite films fabricating, will be discussed in detail. Last but not least, this review will conclude with a summary and some invigorating viewpoints on the key challenges and future developments.

## 1. Introduction

Energy shortage and environmental pollution issues are receiving increasing attention due to the rapid industrialization and excessive consumption of resources by mankind [1,2]. At present, energy supply is mainly dependent on fossil fuels, such as coal, petroleum, and natural gas [3]. The utilization of these fossil fuels is inevitably accompanied by the release of various harmful gases, including carbon dioxide, nitrogen oxides and sulfur oxides etc. [4]. Therefore, developing environmentally friendly and renewable energies to replace conventional fossil fuels is considered a perfect solution to remove these issues, and hydrogen energy is one of the most promising candidates. However, over 90% of hydrogen gas is produced from steam reforming of methane in industry, which limits the development of hydrogen energy because methane is non-renewable energy [5]. In 1972, Fujishima and Honda discovered photoelectrochemical (PEC) H_2_ production from water splitting via using a TiO_2_ cell under ultraviolet (UV) light radiation [6]. Photoelectrochemical (PEC) water splitting by solar energy is generally considered a promising strategy to generate hydrogen gas because both water and solar energy are inexhaustible on the earth.

**Figure 1:**
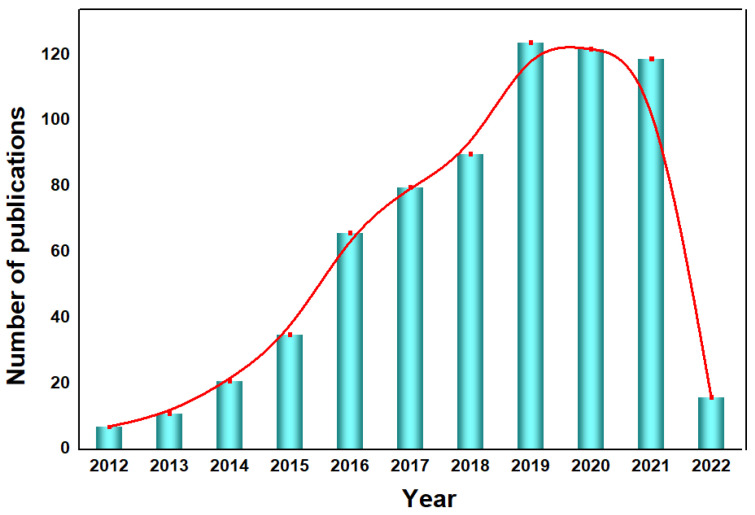
Year-by-year publications of g−CN research on PEC devices, the keywords were set to [“graphitic carbon nitride” and “photoelectrochemical”] (The red line is the trend line). Data are obtained from the ISI Web of Science, dated 17 April 2022.

Carbon nitride-based polymers have attracted widespread attention thanks to their outstanding electronic properties, which have been exploited in numerous applications such as photocatalysis [7,8,9], CO_2_ reduction [10], fuel cells [11], bioimaging [12], and so on. Modified graphitic carbon nitride (g−CN-based films), the focus of this review, can be synthesized and applied for PEC water splitting. Graphitic carbon nitride (g−CN), a metal-free semiconductor, is one of the emerging two-dimensional (2D) materials for PEC. g−CN is widely investigated thanks to its medium band gap structure (ca. 2.7 eV), an appropriate optical wavelength (ca. 460 nm), non-toxicity, high thermal durability (600 °C in air) and chemical durability (against acid, alkali, and organic solvents), and “earth-abundant” nature [13,14,15,16]. Figure 1 illustrate that PEC device applications of g−CN-based films have been soaring since 2016. Nevertheless, it still remains a crucial issue about how to sufficiently probe the unusual features of g−CN for the setout of highly efficient g−CN-based films. The practical application for PEC water splitting is still impeded because it is still difficult to construct a homogenous g−CN film with good crystalline quality on conductive substrates to form photoelectrodes in PEC devices from the viewpoint of the intrinsic layer properties. Furthermore, several key factors still restrict its efficiencies, such as insufficient visible-light absorption, the high recombination rate of charge carriers, short electron diffusion length due to its poor electronic conductivity, and the existence of defects, which act as recombination centers and form a barrier for conductivity. To overcome these issues, various fabrication and modification strategies are applied to improve the PEC performance of g−CN-based films. Enhanced absorption of g−CN-based films is acquired via the construction of π-conjugated polymers, by doping with heteroatoms, or by copolymerizing with different organic monomers [17,18,19]. The conductivity of electrons can be improved by replacing some C=N with C=C bonds, and forming a heterojunction with other semiconductors is utilized to decrease the recombination of photo-excited charges for g−CN materials in PEC applications. In water-splitting PECs, water oxidation (produces oxygen molecules, OER) and water reduction (produces hydrogen molecules, HER) are performed simultaneously but are spatially separated. The oxidation reaction at the photoanode decides the speed of water splitting on account of the sluggish kinetics containing O-H bond dissociation and O-O bond formation [20,21]. Therefore, water oxidation on the semiconductor surface is still considered the bottleneck. To solve this issue, the introduction of a co-catalyst at the g−CN/electrolyte interface is an efficient strategy.

Up to now, several insightful reviews have already focused on the fabrication and modification of the bulk or powder g−CN and their applications in dealing with the energy crisis and environmental problems [3,22]. However, only a handful of reviews can be traced that focused on the synthesis and applications of g−CN films for PEC water splitting. This scenario thus makes it very urgent to offer a critical and relatively comprehensive review of the state-of-the-art advances of g−CN films for PEC water splitting. In the present review, we initially focus on the latest advances in the fabrication, versatile properties, rational modification, and applications of g−CN-based films for PEC water splitting. Firstly, this review will conclude the different types of g−CN photoelectrodes according to their preparation pathways and then the rational modification methods to promote the PEC performance. Last but not least, the major challenges and some invigorating perspectives for future research on g−CN-based film materials are also concluded. We hope that this review will not only offer valuable insight into new g−CN-based film materials but also therefore promote the further developments of g−CN-based films in green and sustainable energy production and storage.

## 2. Water-Splitting Reaction Mechanisms

**Figure 2:**
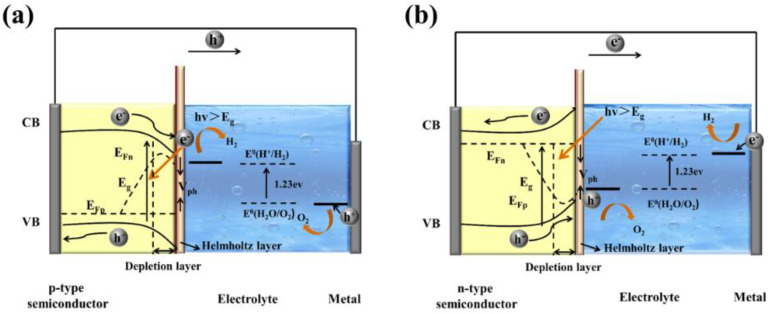
General scheme of a PEC system using semiconductors as (**a**) photocathode and (**b**) photoanode.

Gaining an elementary understanding of photoelectrochemical, charge-separation, and transfer mechanisms is the crucial step in designing photoelectrodes. Figure 2 show structural diagrams of the general photoelectrocatalytic processes for PEC water splitting. There are three primary components of a typical PEC device: (1) a working electrode consists of semiconductor material, a p-type semiconductor as a photocathode (Figure 2a), and an n-type semiconductor as a photoanode (Figure 2b), (2) a counter electrode, and (3) an electrolyte system. It will facilitate the transport of electrons and holes and ensure the separation of hydrogen and oxygen produced on different electrode surfaces. Before contact with the electrolyte, the energy level in the semiconductor is flat. Once in contact with the electrolyte, the different chemical potentials will drive the charge in the semiconductor to the electrolyte until the formation of the built-in electric field. Then, a depletion layer (WD) and a Helmholtz layer (WHL) present at the semiconductor interface and the electrolyte interface, respectively, to form a semiconductor–liquid junction (SCLJ). This charge transfer also gives rise to band bending at the semiconductor interface. For the n-type semiconductors, electrons flow from the semiconductor conduction band to the electrolyte, and the semiconductor surface forms a positively charged depletion layer, resulting in the upward band bending. In contrast, holes flow from the semiconductor to the electrolyte for the p-type semiconductors, which leads to the downward band bending.

In this section, the transport of excitons is discussed. Under a steady illumination, the semiconductor photoelectrode absorbs the photons whose energy is higher than the band gap energy. Then, the electrons become excited from the valence band (VB) to the conduction band (CB), leaving empty states that constitute positive holes in VB. The built-in electric field at the semiconductor/electrolyte interface and the band bending inside the semiconductor separate the electron–hole pairs and consequently generate the photovoltage (V_ph_) and photocurrent. For the semiconductor photocatalysts with a flat band potential above the H^+^/H_2_ energy level, the separation and transport of excitons (electron–hole pairs) do not require external bias to produce H_2_ and O_2_ (self-powered mode). However, the transport of excitons usually suffers from an over-potential across the semiconductor, potential drops at contacts, the resistance of the electrodes, and so on. Therefore, the photovoltage generated by the photoelectrode material is not enough to overcome these losses and provides the energy required for PEC water splitting, and an external bias is needed [23]. Once the charged carriers are spatially separated, they are migrated to the semiconductor photoelectrode surface and initiate the redox reaction for PEC water splitting. In a photoanode, holes are injected into the electrolyte, where they react with OH^−^ to produce oxygen, and the electrons migrate to the counter electrode by a conducting wire that leads to hydrogen generation. In contrast, for photocathodes, the electrons injection electrolyte undergoes a reduction reaction (react with H^+^) to produce hydrogen, while oxygen is produced at the counter electrode. The hydrogen and oxygen evolution reactions of water splitting in different electrolyte environments can be described as follows:

Acidic conditions:

HER: 4H^+^ + 4e^−^ → 2H_2_, E_0_ = 0 V vs. NHE

OER: 2H_2_O + 4h^+^→4H^+^ + O_2_ E_0_ = 1.23 V vs. NHE

Alkaline conditions:

HER: 4H_2_O + 4e^−^ →2H_2_ + 4OH^−^ E_0_ = −0.83 V vs. NHE

OER: 4OH^−^ + 4h^+^ → O_2_ + 2H_2_O E_0_ = 0.4 V vs. NHE

In simple words, the PEC water splitting process can be divided into (1) light absorption by semiconductor, (2) the separation and transportation of electron–hole pairs, and (3) surface redox reactions. Water splitting is an uphill reaction that demands a minimum Gibbs free energy of +237.2 kJ/mol. Theoretically, the minimum band-gap of a semiconductor appropriate for water splitting is 1.23 eV, but a band-gap of approximately 2.0 eV is required due to both thermodynamic energy losses and the over potential required to surmount surface reaction kinetics, which can achieve a theoretical photocurrent density of 14.5 mA cm^−2^ and a maximum solar-to-hydrogen (STH) efficiency of 17.9% under AM 1.5G (100 mW cm^−2^) [24,25,26,27]. To date, n-type semiconductor photoanodes usually exhibit lower photocurrent densities (several mA cm^−2^) under AM 1.5G, which is still much lower than the theoretical values. The semiconductor materials used in overall PEC water splitting must also satisfy specific requirements, which the potential of the conduction band must be more negative than the reduction edge of hydrogen, and the valence band potential must be positive enough to actuate water oxidation (more than +1.23 eV) [28,29,30]. Based on the above discussion, we can infer that a moderate band gap (that is, a sufficient light absorption property), suitable energy level positions, efficient electron–hole excitation and separation, minimal overpotential, long-term stability, and cost-effective are the main requirements for a photoelectrode in PEC water splitting device.

## 3. Electronic Structure of g−CN

**Figure 3:**
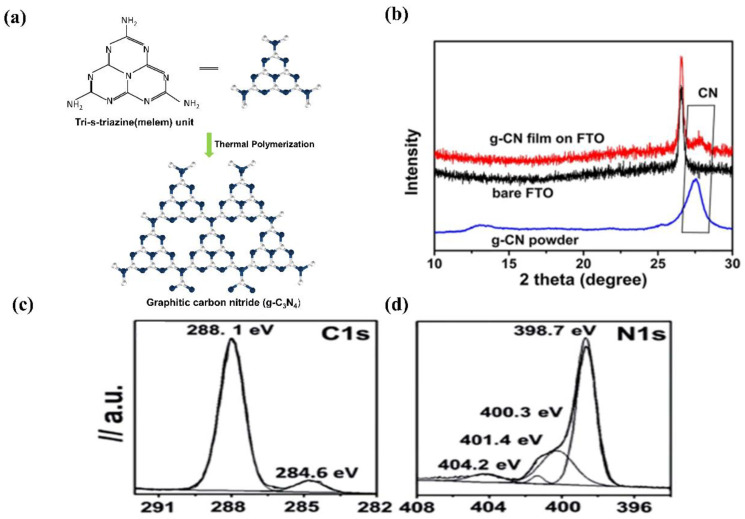
(**a**) Schematic structure of a g−CN constructed from tri-s-triazine units. (**b**) XRD pattern of g−CN film. Reprinted with permission from ref. [34] Copyright 2015 Wiley-VCH. (**c**,**d**) XPS high-resolution spectra of the C1s and N1s core energy spectra of g−CN film. Reprinted with permission from ref. [36] Copyright 2012 Elsevier.

Commonly, g−CN films and g−CN powders possess identically graphitic structures. Ideal g−CN is composed of only C and N elements (C/N ratio is 0.75), showing a band-gap of 2.1 eV. While the synthetic g−CN universally displays a band-gap of 2.7 eV due to incomplete condensation [31]. For the most stable allotrope, the graphitic planes are constituted by triazine C_3_N_3_ (melam) or tri-s-triazine C_6_N_7_ (melem) units connected by planar amino groups (Figure 3a) [32]. However, Tri-s-triazine was regarded as the building block of g−CN because it is a more stable phase at ambient temperature [33]. For pure g−CN, two typical X-ray diffraction peaks observed at 13.04° and 27.251° are attributed to (100) and (002) planes, respectively. The strong peak corresponds to inter-layer diffraction of conjugated aromatic system (002) facet similar to graphite materials; meanwhile, the small peak at (100) is pertinent to the in-plane repeating pattern of tri-s-triazine units formed by hydrogen bonding (Figure 3b) [34]. However, X-ray diffraction (XRD) patterns only offer deficient information due to the low crystallinity of g−CN films. The C1s and N1s X-ray photoelectron spectroscopy (XPS)spectra of g−CN films are exhibited in Figure 3c,d. The C1s spectrum consists of two distinct peaks, the peaks at 284.6 and 288.1 eV correspond to sp^2^ carbon–carbon bonding (C–C) or (C=C), and sp^2^ carbon atoms bonded to nitrogen (C–N=C), respectively. The N1s consist of four peaks. The peaks at 398.7, 400.3, 401.4, and 404.2 eV correspond to sp^2^ hybridized carbon–nitrogen bonding (C–N=C), sp^3^ tertiary nitrogen bonding N–(C)_3_, weak amino hydrogen-bonded functional groups (C–N–H), andπ-excitations (π/π* transition), respectively [35,36].

The band-gap of the laboratory fabricated g−CN is identified to be about 2.7 eV, corresponding to the absorption from the highest occupied molecular orbitals (HOMOs) to the lowest unoccupied molecular orbitals (LUMOs). The HOMO and LUMO levels originated from nitrogen p_z_ and carbon p_z_ orbitals, respectively [37]. This band-gap is large enough to surmount the thermodynamic barrier of the water-splitting, which requires 1.23 eV theoretically. Furthermore, the HOMO/LUMO levels of g−CN straddle the energy levels of water reduction and oxidation, which manifests that g−CN can thermodynamically function as a candidate semiconductor material for PEC [38,39].

Until now, thermal pyrolysis of nitrogen-rich precursors such as cyanamide, dicyanamide, urea, melamine, thiourea, guanidine hydrochloride, trithiocyanuric acid, etc. has been employed to synthesize g−CN. This type of nitrogen-rich precursor has a slight effect on the band-gap structure of g−CN [40]. The flat band potential generated by different precursors was measured using the Mott–Schottky plot, which is −0.6 V vs. RHE for urea [35], −0.8 V for cyanamide and dicyandiamide [41,42], and −0.7 V for melamine [43,44]. The valence band X-ray photoelectron spectroscopy plot, which calculates the energy level difference between the valence band and the Fermi level [45], produced a value of 1.56 V for urea, thiourea and melamine [35,40], and 1.74 V for dicyandiamide [46]. Ragupathi et al. [47] reported a band-gap of 2.89 eV, 2.78 eV, and 2.35 eV for thiourea, urea, and urea with glycine precursor to synthesize g−CN, respectively.

## 4. g−CN Thin-Film Preparation Methods

Numerous synthesis strategies have been developed to prepare g−CN films on substrates, which are classified as thermal vapor condensation (TVC), solvothermal route, liquid-mediated growth, seed growth, electrophoretic deposition, disperse coating, and other deposition methods. Detailed descriptions of the various types of synthesis methods for the construction of g−CN films are provided below. The main advantages and disadvantages of the different synthetic methods are summarized in Table 1.

### 4.1. Thermal Vapor Condensation (TVC)

**Figure 4:**
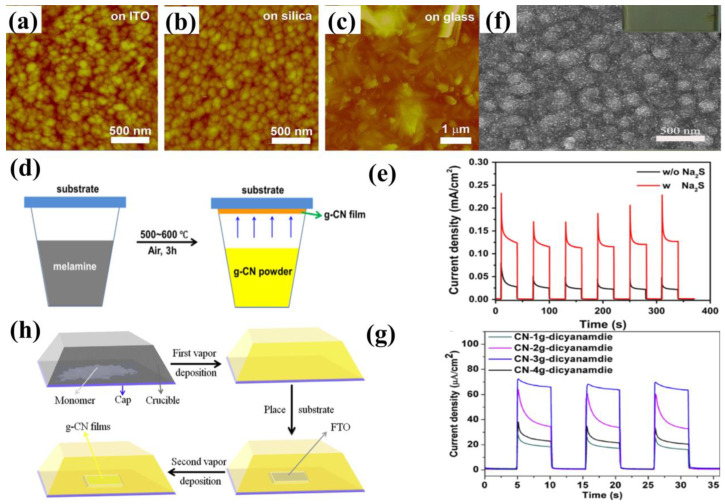
TVC via melamine, illustrating AFM surface morphologies grown on (**a**) ITO glass, (**b**) silica and (**c**) glass, (**d**) TVC growth mechanism, and (**e**) transient photocurrent density of the thin film tested with and without adding Na_2_S at 1.55 V vs. RHE. Reprinted with permission from ref. [48] Copyright 2015 Elsevier. Two-step TVD procedure utilizing dicyanamide: (**f**) SEM images of the g−CN electrodes (the inset is the digital image), (**g**) the corresponding transient photocurrent density in 0.1 M Na_2_SO_4_ at 1.23 V vs. RHE, and (**h**) two-step TVC growth mechanism. Reprinted with permission from ref. [49] Copyright 2017 Elsevier.

The TVC setups for the deposition of g−CN thin film are shown in Figure 4d,h. Owing to the different deposition sequences, TVC can be categorized into two forms: the one-step process and the two-step process. In a one-step TVC procedure (Figure 4d), the substrate is placed on top of a reactor. Taking nitrogen-rich precursor for an example, the precursor vapor generates if the temperature is higher than its sublimation point (above 300 °C). Then, the vapor inside the container will deposit on the substrate surface when the temperature reaches the condensation temperature (500–600 °C) of the precursor, resulting in the nucleation and growth of g−CN thin film [48]. While for the two-step TVC procedure, g−CN is condensed and deposited on the inner wall of the container upon the first vapor deposition. Subsequently, the substrate is placed on top of a reactor. The g−CN attached to the container is vaporized again and grown on the g−CN thin film on the substrate surface [49]. To date, g−CN thin film has mainly been prepared by TVC with different precursors, such as melamine [50], dicyanamide [49], thiourea [51], urea [52], and cyanamide [53], and the bulky g−CN is always utilized. Bian et al. discovered that melamine, compared with dicyandiamide, thiourea, and urea, shows the highest photocurrent density for the PEC system in a one-step TVC procedure [48]. The g−CN film fabricated with dicyanamide shows a photocurrent density of up to 63 μA cm^−2^ at the bias of 1.23 V versus RHE without a sacrificial reagent in a two-step TVC procedure (Figure 4f–g) [49].

The dependency of the properties of g−CN films on the influence factors, including deposition temperature, altering the substrate, precursor amount, and sacrificial reagent, will be compared and discussed. The vapor deposition method has been widely explored for depositing uniform and dense g−CN films on various substrates, such as indium-doped tin oxide (ITO) glass, fluorine-doped tin oxide (FTO) glass, silicon slice, quartz glass, and glass. Bian et al. discovered that the morphology of the g−CN film is substrate-dependent, grown on ITO glass and silica are similar to that of on FTO glass; they are continuous films with many nanoparticles on the surface. However, inhomogeneous particles and nanorods formed on glass (Figure 4a–c) [48]. The sacrificial reagent is able to not only facilitate hole consumption, which can decrease the recombination of electron-hole pairs but also is significantly affected by the holes in the energy levels where they cannot react with OH^−^. The g−CN film prepared with melamine shows a photocurrent density up to 30 μA cm^−2^ versus RHE without sacrificial reagent under one sun illumination at 1.55 V. When sacrificial reagent was added to electrolytes, the highest photocurrent density reached up to 120 μA cm^−2^ (Figure 4e) [48]. Both the thickness and the optical light absorption of the films increase with the increasing mass precursor. The increased film thickness reduces the transfer resistance of electron-hole pairs between film and electrolyte interfaces, but a high mass precursor makes the films partly peel off [49]. Based on the experiment analysis, an increased deposition temperature is beneficial to enhance the optical light absorption and increase the thickness of the films. This indicates that the light absorption alongside the films shifts towards red-shift as the deposition temperature increases, which is very consistent with the absorption alongside the change of the bulk g−CN [54,55]. However, the high process temperature (above 650 °C) makes substrates largely deform [48], decreasing the uniformity and the PEC performance.

**Figure 5:**
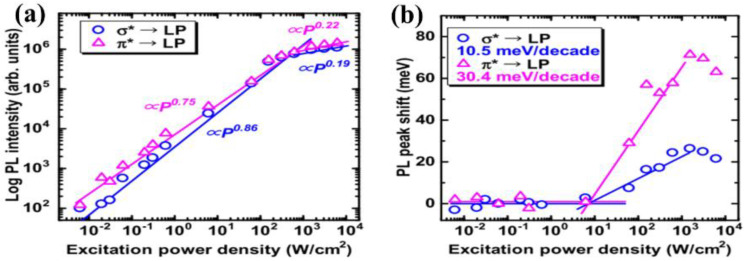
Excitation-power dependence of (**a**) the integrated PL intensities and (**b**) PL peak shift for σ*-LP and π*-LP transition paths of the thin film. Reprinted with permission from ref. [56] Copyright 2019 Japan Society of Applied Physics.

The luminescence mechanism of the g−CN thin film was reported by Urakami et al. [56], who successfully realized high-quality g−CN films on different types of substrates, such as SiO_2_, HfO_2_, and c-plane sapphire through TVC at 600 °C. A crystalline g−CN thin film can be deposited on both non-crystalline and crystalline surfaces by employing an amorphous-like buffer layer. The bonding states and stoichiometric chemical composition (N/C ratio was 1.36–1.39) are close to those of ideal g−CN. From the dependence of the photoluminescence (PL) intensity and peak shift on the excitation–power–density, electron injection from the sp^3^ C–N σ to the sp^2^ C–N π conduction band was strengthened under the above excitation condition (E_ex_ > E_g_ σ) (Figure 5a,b).

Based on the above analysis, TVC is considered a very simple, convenient, and effective method to prepare g−CN films. In addition to morphology, the thickness and structure of the g−CN film can easily be controlled. However, the number of precursors that can be utilized is restricted. Other frequently reported precursors fail to form g−CN films on solid substrates [48]. As is well known, NH_2_ groups play an essential role in nucleation. However, a lack of one NH_2_ group in each precursor molecule is proven to fail to realize high-quality g−CN thin film [53].

### 4.2. Solvothermal Route

**Figure 6:**
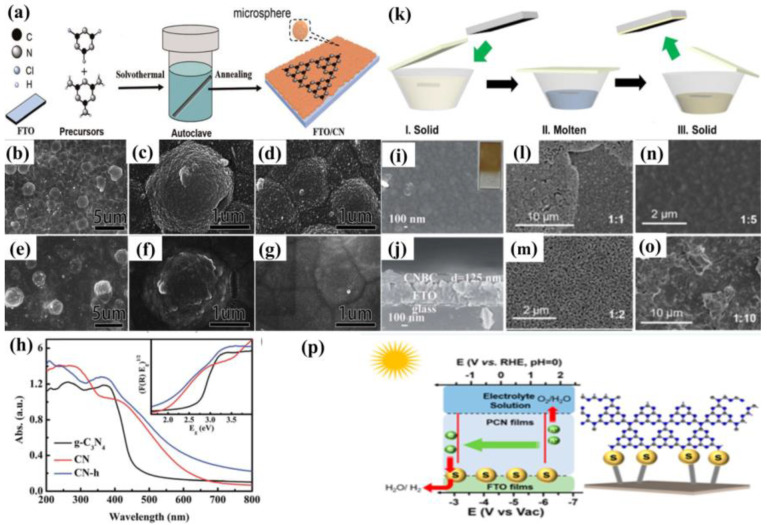
(**a**) Schematic illustration of the solvothermal synthesis route. Solvothermal approach utilizing melamine and cyanuric chloride: SEM images of (**b**–**d**) g−CN sample before annealing and (**e**–**g**) g−CN sample after annealing and (**h**) UV-vis absorption spectra (the inset shows the Tauc plots). Reprinted with permission from ref. [57] Copyright 2016 Royal Society of Chemistry. Solvothermal approach using cyanuric chloride and cyanuric acid: SEM images of the top view (**i**) and cross-section (**j**) of annealed g−CN thin film (the inset is the digital image). Reprinted with permission from ref. [58] Copyright 2017 Royal Society of Chemistry. (**k**) Liquid mediated growth mechanism, (**l**–**o**) SEM images of the structured PCN films with different molar ratios (the mixture of melamine and trithiocyanuric acid) after condensation at 500 °C for 4 h, and (**p**) the growth mechanism of the PCN films by combination of sulfur and non-sulfur mixtures and mechanism for water splitting. Reprinted with permission from ref. [59] Copyright 2014 American Chemical Society.

The solvothermal route is a procedure to arrange the covalent bond between the molecular precursors and the substrate surface [57,58]. It includes two essential steps (Figure 6a): Firstly, the substrate is placed in an organic solvent that is filled with nitrogen-rich precursor solutions. Subsequently, the g−CN films were annealed for the purpose of further polymerization and enhancing the contact between film and substrate.

Using the solvothermal method with post-annealing, g−CN films can be grown on different substrates, such as FTO [57,58], glass [58], and TiO_2_/FTO [58]. Xie et al. fabricated g−CN films on FTO via simply dissolving cyanuric chloride and melamine in acetonitrile, as described in Figure 6a. The solutions were maintained at 180 °C for 24 h, and the g−CN film followed the Stranski–Krastanov growth mode. A transition from the two-dimensional layer-by-layer mode (shorter than 12 h) to three-dimensional micro-spheres stacking mode was observed owing to the changes in the surface energy of substrates and the properties of solutions. However, this process can hardly form a complete tri-s-triazine structure g−CN film because of insufficient kinetic energy. An annealing process, annealed in N_2_ at 520 °C for 1 h, for example, plays a key role in forming a tri-s-triazine structure. The interfaces of microspheres and nanoparticles become vague after a post-annealing treatment, manifesting the thermal cross-linking and condensation sequentially (Figure 6b–g). The obtained g−CN films showed higher activity in PEC performance of this technique, with photocurrent densities of 3.5 μA cm^−2^ at 1.23 V vs. NHE produced, compared with traditional paste-prepared g−CN. The improvement was mainly attributed to the following two factors. Firstly, the presence of covalent linkage between g−CN and the substrate generates synergistic effects. Secondly, a red shift from approximately 460 nm to 620 nm is accompanied by the diminished band gap of 2.0 eV, narrower than the 2.7 eV of the conventional g−CN (Figure 6h) [57].

Recently, a significant improvement was achieved by Gu et al. with a facile solvothermal method. Using cyanuric chloride and cyanuric acid as precursors, highly crystalline g−CN thin films were obtained on different substrates with post-annealing at 450 °C. In consequence, the photocurrent densities of 10 μA cm^−2^ at 1.23 V vs. RHE are produced. The resulting g−CN film exhibits enlarged conjugating structures of tri-s-triazines and s-triazines, allowing uniform and continuous interfacial contact (Figure 6i,j). Moreover, when the film was grown on the surface of anatase TiO_2_, the photocurrent densities were further enhanced owing to vectorial charge transfer induced by the layer-by-layer configuration [58]. This work manifests the vital effects of morphology and structure on the properties of carbon nitride films for PEC water splitting.

In addition, an effective tactic for controlling the thickness and density of g−CN films can be achieved by changing the growth time, precursor concentration, and post-heating temperature. The improvement of PEC performance was attributed to the presence of covalent linkage due to introducing either cyanuric acid or cyanuric chloride, which enhances the charge carrier transportation. Furthermore, both studies reported a diminished band gap due to improved π-electron delocalization of the conjugated structure, widening the light absorption range [57,58]. However, the obtained g−CN films for PEC water splitting were not as good as we expected, seemingly aroused by the rich surface defects of carbon nitride films. Developing new solvents and combining multiple precursors to tailor g−CN is particularly important.

### 4.3. Liquid-Mediated Growth

To obtain uniform, transparent, and reproducible g−CN solid thin films, the precursor in the liquid reaction system should be granted priority because liquid-mediated growth provides more intimate contact with the substrate than its common solid-phase counterpart.

The process of liquid-mediated growth is shown in Figure 6k [59]. Firstly, the substrate is fixed to the bottom of a crucible that is filled with nitrogen-rich solid precursors. Upon heating, this solid precursor melts into the liquid phase, obtaining a chummy contact with the surface of the substrate. Finally, post-annealing condensates the liquid phase and eventually turns it into a solid film. The obtained film is continuous, stable, and hand-in-glove adhered to the surface of various substrates, including FTO, ITO, glass, metal foil, and TiO_2_, providing great potential for the fabrication of high-performance devices. The supramolecular pre-assembly strategy has attracted extensive attention because its precursors show an identical chemical structure to g−CN and effectuate liquid-mediated growth [60]. Shalom et al. [61] utilized this strategy to form ordered structures of g−CN via hydrogen-bonded cyanuric acid-melamine supramolecular complex. The solid complex locals between two desired substrates were post-annealed to form homogeneous and comparatively organized g−CN rods. Besides its hydrogen bonds, the complex appears to possess free hydroxyl and amine groups which can be grown on varying substrates (glass, FTO, TiO_2_, ZnO, etc.) with diverse morphologies of g−CN. The electrode demonstrated current densities could reach up to 0.8 mA cm^−2^ at 0.6 V (vs. RHE) in neutral media.

In another similar work by Xu and co-workers, [59] the obtained g−CN film exhibited continuous porous network morphology on various substrates since no template was utilized during the fabrication. The mechanical robustness of the g−CN films fabricated utilizing a liquid-mediated pathway was very high, obtaining thin films that were not peeled off via sonication in water for 1 h. Moreover, g−CN films fabricated through supramolecular aggregates improve the optical and electronic properties because of their enhanced structure intactness. Inspired by these facts, Xu and co-workers [62] presented a liquid-mediated approach to preparing continuous phenyl-modified carbon nitride films via combining molten sulfur and 2,4-diamino-6-phenyl-1,3,5-triazine–cyanuric acid complexes. The photocurrent density of the prepared phenyl-substituted g−CN photoelectrode was up to 60 μA cm^−2^, which was 20-fold higher than that of non-sulfur-processed. The high-temperature molten sulfur integrates phenyl groups into the thin film effectively to enhance the conductivity and charge transfer, which is beneficial to improving the PEC efficiency. The intermediate state plays a crucial role in the monomer’s preorganization for the liquid-phase thin-film growth, owing to its influence on the supramolecular interactions [63]. Aside from film quality and intermediate state, the insufficient binding between the functional films and conductive substrate is also considered a major issue that hinders Polymeric carbon nitride (PCN) films for solar water splitting. To overcome this challenge, Fang et al. proposed a combination precursor of sulfur and non-sulfur complexes for the preparation of polymeric carbon nitride films [64]. It is found that sulfur exists on the PCN/FTO glass interface, which not only initializes the formation of the PCN films but also assists the charge migration between films and the substrate (Figure 6l–p). A preeminent photocurrent density of 100 μA cm^−2^ was achieved at 1.23 V (vs. RHE) under AM 1.5 illumination without sacrificial reagents. Inspired by the aforementioned method, the Ni chloride salt molecules were also introduced into the cyanuric acid and 2,4-diamino-6-phenyl-1,3,5-triazine supramolecular aggregates. The band gap and catalytic properties of the obtained g−CN films could be regulated via altering the Ni amount, and the PEC properties were prominently improved. The Ni atoms within the carbon nitride layers significantly extend the light absorption range and enhance the charge transfer properties [65].

### 4.4. Seed Growth

**Figure 7:**
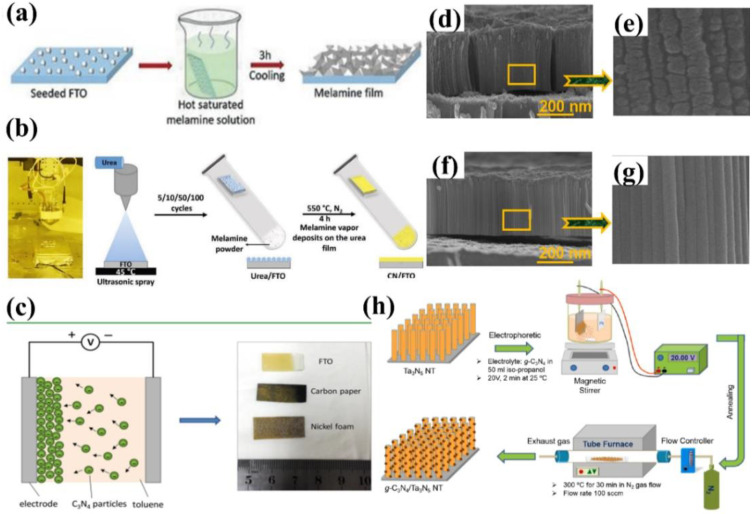
(**a**) Schematic illustration of the synthesis of melamine film, reprinted with permission from ref. [66] Copyright 2018 John Wiley and Sons Ltd. (**b**) Digital photo of the spray coater system, Reprinted with permission from ref. [68] Copyright 2021 Wiley-Blackwell. (**c**) A diagram illustrating the process of electrophoretic deposition, digital image of the deposited g−CN on FTO glass, carbon paper, and nickel foam, Reprinted with permission from ref. [71] Copyright 2016 American Chemical Society. (**d**–**g**) Cross-sectional views of FE-SEM and Magnified view of the Ta_3_N_5_NT/Si and g−CN/Ta_3_N_5_NT/Si films. (**h**) Representative schematic diagrams to represent the experimental procedures and methods of the g−CN. Reprinted with permission from ref. [72] Copyright 2021 Elsevier.

Seed growth has been developed to fabricate g−CN films, and it includes three main steps: seeding the substrate, soaking the seeded layer into a hot supersaturated precursor solution, and calcination. The seeds are protected by the supersaturated precursor solution to prevent dissolution, which acts as nucleation sites for the crystallization of precursor from the solution. Upon cooling, precursor crystals spontaneously grew on the seeded substrate surface to form a well-covered film. Upon calcination of the as-prepared film, a g−CN layer was synthesized on the substrate. Shalom et al. [66] grew highly ordered carbon–nitrogen films via the seeded crystallization of carbon–nitrogen monomers, leading to a photocurrent density of 116 μA cm^−2^ at 1.23 V versus RHE (Figure 7a). A similar approach was used for the fabrication of g−CN-based films on conductive carbon paper [67]. Recently, Shalom et al. [68] developed a new approach to growing thick g−CN layer films via a spray-coated seeding layer composed of CN monomers, leading to an impressive photocurrent density of 300 μA cm^−2^ at 1.23 V versus RHE in the presence of a hole scavenger (Figure 7b), implying the high scalability potential of this method for the flexible material and device applications.

### 4.5. Electrochemical Deposition

To date, there are two representative routers of electrochemical deposition, including (1) electrodeposition (ED) in the involvement of ionic precipitation [35,69,70] and (2) electrophoretic deposition (EPD) based on the adsorption of charged granules onto externally biased electrodes [45,71]. In the last few years, there has been growing research interest in the fabrication of g−CN-based photoelectrodes by utilizing electrochemical deposition methods. Xu et al. [71] presented a mild pathway to construct a uniform g−CN film through the EPD approach under an anodic bias of 200 V, which can be successfully grown on different substrates such as FTO and carbon paper as well as nickel foam with complex 3D geometries. Toluene was used as a solvent owing to its large electrochemical window. The films deposited on carbon paper proved a higher photocurrent response of 12 μA cm^−2^ compared with 1 μA cm^−2^ on FTO under zero bias. The photocurrent was further improved significantly to 65 μA cm^−2^ by doping with barbituric acid. Therefore, tailored construction of these modified carbon nitride layers led to efficient contact with electrolytes due to the high surface area of 3D carbon paper and enhanced light-harvesting and conductivity via heteroatom doping (Figure 7c). Recently, Kang et al. designed the g−CN grown on Ta_3_N_5_ nanotube/Si substrate by electrophoretic deposition to enhance PEC performance (Figure 7d–h) [72].

### 4.6. Disperse Coating

In spite of the fact that g−CN powder has been extensively and deeply researched, the preparation knowledge of g−CN film is still in its infancy. Recently, disperse coating strategy from colloid sources has also been reported extensively owing to its extraordinary features, including facile and cost-effectiveness. It has been applied to the deposition of carbon nitride thin film on various substrates via conventional filming technologies, such as spin coating [73], silk-screen printing [74], drop-casting [75], dip casting [76], doctor blade coating (DBC) [77], and Sol-gel process [42]. The uniformity and performance of the g−CN films mainly depend on the influenced various parameters of g−CN colloids in terms of chemical bonding and size of particles [42].

In order to fabricate the uniform g−CN thin films, the principal requirement is to obtain a stable dispersion in appropriate solvents such as alcohol, water, and methanol. Several researchers gained g−CN dispersions in water [78,79] or the combination of alcohol and water [80,81]. Nevertheless, the existence of water has always been restricted to obtain uniform thin films due to its high boiling point and surface tension. Furthermore, due to the poor solubility of g−CN powder in most solvents, the fabricated g−CN films usually present poor physical contact with the substrate as well as boundary defects, which results in a weak photocurrent response of less than 1 μA cm^−2^ [41,82,83].

As we know, exfoliation of two-dimensional structures shows abundant surface active sites and effectively captures the visible light compared to bulk g−CN [79,84,85]. From the practical application viewpoint, the alteration of bulk g−CN by two-dimensional g−CN nanosheets with a unilamellar structure via adopting a physical modification process (ultrasound-assisted or hydrolysis), chemical exfoliation with different solvents (acids, salts, bases, etc.), and physicochemical methods (heating and hot water) are widely used. Zhang et al. [77] demonstrated that the protonation of g−CN using strong acid (HNO_3_) results in a sol, which allows the fabrication of films through facile dip coating. The melon units stack together through hydrogen bonds between the nitrogen atom and NH/NH_2_ group, which in principle can be used as proton acceptors. To the best of our knowledge, a stable g−CN thin film with high photocurrent density not only benefits from the post-annealing process but, more importantly, should preserve its unique physicochemical features after robust HNO_3_ etching. As a result, there is great potential for applying the sol-gel technique for carbon–nitrogen PEC application, which shows enhanced PEC response compared to powder-based thin films.

Despite significant advances in the exfoliation of g−CN powders, the finding of ultrathin or monolayer g−CN nanosheets through strong acid treatment or chemical exfoliation undergo slight defects due to the fact that the preparation process is not environmentally friendly. Therefore, scientists suggested an urgent demand to obtain a stable and environmentally friendly dispersion for the preparation of g−CN films, which are solvents. Safaei et al. [86] demonstrated that methanol is preferable for layered g−CN due to the short drying time and significantly low surface tension. Inspired by this idea, a very promising work was reported by Mohamed and co-workers [87], who showed a simple synthetic method for the fabrication of a thinner layer by layer from bulk g−CN by employing a slow direct low thermal treatment (40 °C, 24 h) with ultrasonication, which was obtained as the highly stable g−CN suspension in a methanol solution. The exfoliation of nanosheets from bulk g−CN can form a uniform layer of g−CN thin films via using a spin-coating method followed by an appropriate post-annealing temperature at 350 °C. The obtained g−CN thin film presents a more negative conduction and valence band potentials as well as a much rougher surface (Sa roughness: 14.58 nm), which are generally helpful to PEC performance. Moreover, the photocurrent responses of the prepared g−CN films reached up to 10.21 μA cm ^−2^ at 1.23 vs. (Ag/AgCl), which was two times higher than that of bulk g−CN.

The post-annealing temperatures also play an important role in the morphology and the chemical bonding of the g−CN thin film that ultimately influences the PEC efficiency. Zheng and co-workers [88] investigated that post-annealing transforms the hollow carbon nitride nanospheres into the triazine unit. Mohamed et al. [52] found that post-annealing improves the chemical bond structure and morphology of g−CN fabricated by the spin-coating method. In addition, there is a direct relationship between post-annealing temperature and band gap. The band gap reduces from 2.79 eV to 2.71 eV by increasing the post-annealing temperature from 150 ℃ to 350 °C, while it increases from 2.71 eV to 2.85 eV at higher temperatures (350 °C to 500 °C), the energy band gap of the g−CN thin film was enlarged. This phenomenon is ascribed to the quantum confinement effect and the shift of the CB and VB in opposite directions [88].

Whereas using the supramolecular assembly affords thin dense layers, applying a paste made up of supramolecular precursors in dispersant (such as ethylene glycol) enabling hydrogen bonds leads to porous layers of controllable thickness. It is noteworthy that the carbon–nitrogen monomers can connect with triazine derivatives (e.g., barbituric acid and cyanuric acid) to form supramolecular paste via hydrogen bond interactions [22]. Changing the supramolecular precursors can control the elemental ingredient as well. Therefore, this method enables the application of the knowledge to progress from powder-based g−CN synthesis to g−CN-based electrodes. Based on the supramolecular pre-assembly approach, an ameliorative work based on g−CN films was reported by Shalom and co-workers, who presented a general and simple pathway for the construction of highly porous and large-scale g−CN films through forming a supramolecular paste, which can be successfully grown on different substrates using the doctor blade technique [89]. g−CN has a 2D layered structure connected by van der Waals forces. When it is stripped into multilayer or single-layer nanosheets, unique chemical and physical properties are produced to further improve PEC efficiency, including (1) larger specific surface area to increase the density of the reactive center, (2) reduced thickness to reduce the carrier migration distance and effectively inhibit carrier recombination, and (3) unique two-dimensional flexible planar structure to enhance compatibility with heterojunction construction, cocatalyst modification, vacancy, and other modification strategies.

### 4.7. Other Deposition Methods

**Figure 8:**
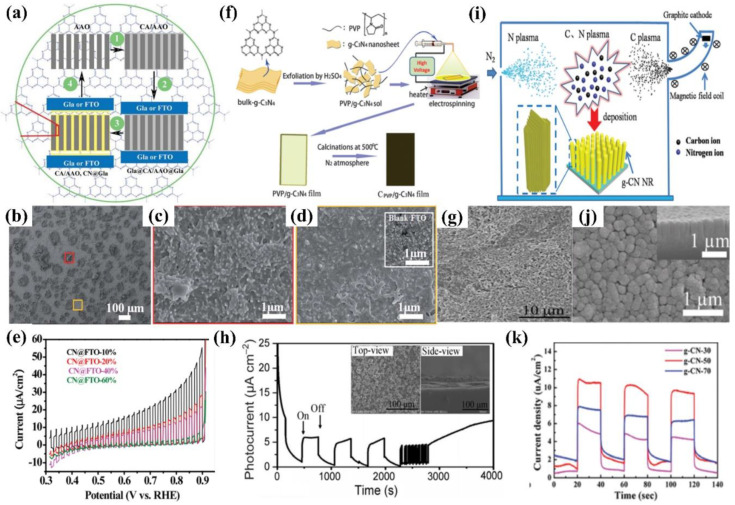
(**a**) Schematic illustration of printing g−CN thin films on “sandwiching” substrates (AAO template growth mechanism), (**b**) the surface morphology, (**c**) magnified image of the selected box area (red) in (**b**) displaying that the nano−islands, (**d**) magnified image of the selected box area (yellow) in (**b**) displaying the detailed microstructures (the inset is the bare FTO image), and (**e**) LSV photocurrent density in 0.2 M Na_2_SO_4_ at 0.86 VRHE. Reprinted with permission from ref. [90] Copyright 2015 Wiley-Blackwell. (**f**) A diagram illustrating the process of electrospinning mechanism, (**g**) top−view SEM image of the CPVP/g−CN film on ITO glass, (**h**) time dependence of the photocurrent density of CPVP/g−CN in 0.05 M Na_2_SO_4_ at a 0.5 V vs. SCE (the insets are top-view and cross-section SEM images of the film after testing). Reprinted with permission from ref. [77] Copyright 2017 Wiley-VCH. (**i**) Schematic illustration of VMFAIP system used for fabrication of g−CN NRs on FTO glass, (**j**) SEM images of the top-view and cross-sectional (inset), (**k**) transient photocurrent response under different target currents in 0.5 M Na_2_SO_4_ at 1.23 V vs. RHE. Reprinted with permission from ref. [93] Copyright 2018 Royal Society of Chemistry.

Some other ex situ methods have also been developed to fabricate g−CN electrodes successfully, including anodic aluminum oxide (AAO) membrane deposition [90], direct growth [61], vacuum filtration deposition [91,92], electrospinning (ESP) [77], vacuum magnetic filtered arc ion plating (VMFAIP) [93], ionothermal (molten salt) deposition [94], radio frequency magnetron (RFM)−based sputtering [95], and so on. A microcontact-printing process was reported by Liu et al., who fabricated g−CN nanostructures by injecting cyanamide into an anodized aluminum oxide (AAO) membrane sandwiched between two substrates and subsequently calcinating at 550 °C for 4 h (Figure 8a). The AAO is not a versatile template but a guide for gas-phase precursors to reach and react with the sandwiching substrates. The film thicknesses can be simply modulated by varying the concentration of cyanamide. The photocurrent density can reach about 30 μA cm^−2^ at 1.23 V vs. RHE (Figure 8a–e) [90].

Electrospinning as a practicably proven method is also used to fabricate the PEC photoanode, in which the ITO substrate is coated with prepared polyvinylpyrrolidone (PVP)/g−CN sol and annealed at 500 °C. The thickness of the films can be modulated by varying the electrospinning time and actually controlling the PEC performance. Furthermore, the photoelectrode demonstrates a stable photocurrent performance (6.64 μA cm^−2^) for 4000 s with no degradation (Figure 8f–h) [77]. Wang et al. developed a vacuum magnetic filtered arc ion plating (VMFAIP) approach, in which the carbon plasma emitted from a graphite cathode was deflected by a magnetic field and combined with N_2_ to generate C–N compound on the FTO substrate. The resulting g−CN nanorod (NR) arrays eliminated the intralayer hydrogen bonds owing to the utilization of carbon and nitrogen sources alone. The change of the graphite target current allowed different lengths of the g−CN nanorod arrays from 550 nm to 1.1 µm. The gas-based approach via the VMFAIP system enabled the g−CN to form heterostructures easily with, including but not limited to, TiO_2_ nanorod arrays (Figure 8i–k) [93]. Ladva et al. [94] synthesized g−CN thin films with a bandgap of approximately 1.73 eV by an ionothermal (molten salt) approach using dicyandiamide, KBr, and LiBr mixture precursors. Lou et al. [96] demonstrated a g−CN layer on the substrate by embedding g−CN in a matrix conductive polymer binder (PEDOT-PSS [96,97,98], Nafion [57,99,100], and others) [101]. Compared with Nafion, the g−CN film blended by PEDOT-PSS showed superior PEC activities because the PEDOT-PSS binder is a wonderful conducting polymer and can act as a hole-transport layer. Recently, Tejasvi et al. [95] synthesized stoichiometric g−CN thin films by radio frequency magnetron sputtering using a g−CN pellet as a target. Thin-film samples can be deposited on the borosilicate glass and TiO_2_ nanotube array in Ar and N_2_ plasma media. The PEC studies summarized that the g−CN/TiO_2_ formed under Ar plasma (~290 μA/cm^2^ at 0.9 V) obtained a higher current density as compared to that under N_2_ plasma (~160 μA/cm^2^ at 0.9 V).

Although various techniques have been employed to prepare g−CN electrodes, g−CN films prepared by ex situ methods mostly show poor photocurrent density (several μA cm^−2^), which can be ascribed to the uneven coverage and the weak adhesion of g−CN on the substrates [83]. In contrast, the g−CN films in situ synthesized on various conductive substrates by a bottom-up route generally exhibit good photocurrent density (10–120 μA cm^−2^) [66]. Shalom et al. [66] grew highly ordered g−CN films via the seeded crystallization of melamine monomers, leading to a photocurrent density of 116 μA cm^−2^ at 1.23 V versus RHE under AM 1.5G in 0.1 M KOH aqueous solution. This is the best value reported for pure g−CN electrodes without a sacrificial agent. Enormous space still exists for achieving a theoretical photocurrent density of 14.5 mA cm^−2^ and a maximum solar-to−hydrogen (STH) efficiency of 17.9% under AM 1.5G (100 mW cm^−2^) [24,25,26]. The various types of synthesis methods for the construction of g−CN films with the corresponding photocurrent comparison are summarized in Table 2. In addition, in order to produce g−CN with a uniform texture, casting g−CN embedded in a conductive polymer matrix tactics is highly worth considering. The method has two-fold advantages: it improves the charge transfer in the film and enhances the casting homogeneity. Despite this, enormous space still exists for devising efficient tactics to fabricate homogenous electrodes. 

## 5. Functionalization of g−CN Photoelectrode

### 5.1. Heteroatoms Doping

When pure g−CN films are employed as photocatalysts, their practical applications are still hindered by limited theoretical photocurrents. The p–p* transitions in the conjugated aromatic system of g−CN result in a band gap of 2.7 eV, suppressing the absorption above 460 nm [75]. Doping of additional atoms and impurities into the g−CN matrix is considered an effective strategy to ameliorate structural defects and distinctly tune its physicochemical properties, including extending visible-light absorption scope and regulating redox potentials to further promote the PEC properties. There are two main types of elemental doping that will be explored in this section, namely nonmetallic elements doping and metallic elements doping. 

**Figure 9:**
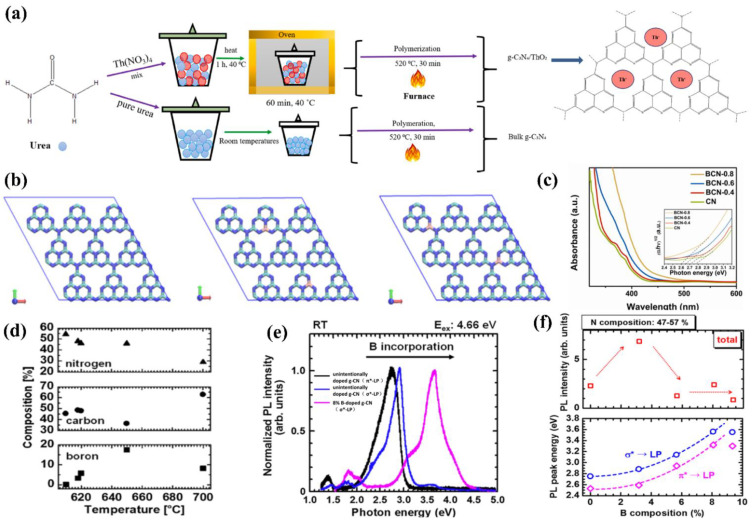
(**a**) Schematic illustration of the formation process. Reprinted with permission from ref. [104] Copyright 2021 Elsevier. (**b**) Top-views of super-cell 3 × 3 pure g−CN (left) and B-doped g−CN with two C substituted by B at corner (middle) and bay (right) sites in a lattice with constants of a = b = 21.04 Å, c = 20.0 Å, and α = β = 90°, γ = 120° by DFT optimizations. The green, blue, and pink colors represent C, N, and B atoms, respectively, and (**c**) the UV–vis absorption spectra (the inset shows the Tauc plots). Reprinted with permission from ref. [106] Copyright 2018 Elsevier. (**d**) Substrate temperature dependence of compositions, (**e**) typical RT-PL spectra for B-doped g−CN films with various compositions, the black line is the a peak energy corresponds to unintentionally doped g−CN films (π*-LP), the blue line is the a peak energy corresponds to unintentionally doped g−CN films (σ*-LP), and the pink line is the a peak energy corresponds to B-incorporated g−CN with a composition of 8.0% (σ*-LP) and (**f**) room temperature integrated PL intensity and PL peak energy depended on B composition. Reprinted with permission from ref. [110] Copyright 2020 Wiley-VCH.

Qi and co-workers [102] prepared Ag-doped g−CN films (Ag/g−CN) via a liquid-based reaction process employing AgNO_3_ as the Ag dopant. The AgNO_3_ strongly affects the PEC efficiency of the Ag/g−CN films. The optimized photocurrent density was as high as 6.40 μA mm^−2^ for the Ag/g−CN films (molar ratio of 1: 10), which was approximately 6.8−fold higher than that of a pristine g−CN film electrode. This is due to the efficient charge transfer and improved light-harvesting property of Ag-doped g−CN film. Apart from Ag, Karimi-Nazarabad and co-workers [103] developed a new type of Pd-doped g−CN photoanode via an electrophoretic deposition (EPD) strategy. A Pd-doping amount of 2 at% into g−CN exhibits a transient photocurrent density of 79.2 μA cm^−2^, which is notably higher than those of Ag/g−CN and pristine g−CN films. Additionally, the photoelectrocatalytic oxygen evolution performance is improved due to the facilitated transfer efficiency of photo-generated charge carriers, which can be ascribed to the deeper band bending and the remarkable trapping capability for electrons. Recently, doping engineering with Thorium Nitrate (ThO_2_) during g−CN polymerization altered the chemical bonding, with the improved PEC activity (Figure 9a) [104]. The Sr-doped g−CN showed enhanced PEC activity [105].

In addition to the metallic atom-doping mentioned above, the incorporation of non-metallic atoms such as B, C, and S into the g−CN framework was also studied. Compared with metal doping, the superiority of nonmetal doping is distinct due to its capability of reserving the nonmetallic properties of g−CN.

Huang and co-workers [106] presented a facile TVC method to prepare B-doped g−CN films using boric acid as the B source. The XPS analysis clearly confirmed that the B atoms were successfully incorporated into the g−CN framework and replaced the C composition partially (Figure 9b). The B doping effectively reduced the band-gap by raising the valence band and prolonging the charge carrier lifetime by altering spatially orbital distributions. Optical band-gap energies decreased from 2.77 to 2.60 eV with the increasing boric acid content in the precursor (0 to 0.8 g), extending the photon absorption edge (Figure 9c). The photocurrent density of the optimized B-doped g−CN films was as high as 55 μ Acm^−2^ at 1.23 V vs. RHE, which was four-fold higher than that of pristine g−CN films. The type of species supplied from the precursors and the pyrolysis temperature has a slight effect on the chemical compositions of compound semiconductor alloys [107,108,109]. Therefore, to control additional atoms composition and band-gap energy, the influence of pyrolysis conditions on additional atoms incorporation into the C–N network should be expounded. Inspired by these facts, Urakami and co-workers [110] reported a simple thermal chemical vapor deposition (CVD) route to prepare the B-incorporated g−CN film by employing ammonia borane (H_3_N–BH_3_) as the B source. It was also noted that the pyrolysis temperature had an intimate connection with the compositional ratios, and the preferable temperature for B doping ranged from 618 °C to 650 °C (Figure 9d). Importantly, a shift of the PL peak from 2.7 eV to 3.60 eV was manifested by B-incorporation with a composition of 8.0%. The PL peak energies for σ*−LP and π*−LP shift routes were in accordance with the quadratic function, manifesting that the band-gap bowing appeared as in orthodox compound semiconductor alloys (Figure 9e,f).

**Figure 10:**
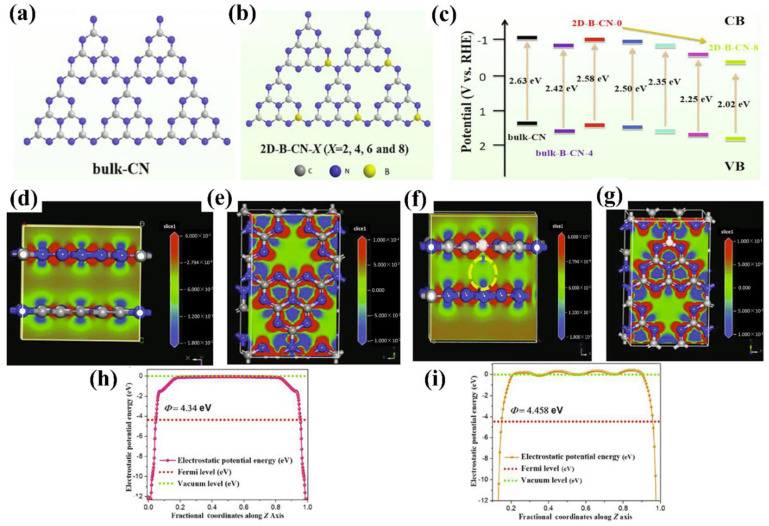
(**a**,**b**) The detailed structures of bulk-CN and 2D B doped g−CN (2D-B-CN) samples. (**c**) schematic energy level diagrams. (**d**,**e**) Electron density difference (EDD) plots and (**h**) work function of bulk-CN. (**f**,**g**) EDD plots and (**i**) Work function of 2D-B-CN (C, N and B atoms are labelled in gray, blue, and white, respectively). Reprinted with permission from ref. [112] Copyright 2022 Elsevier.

In addition, a novel two-step surface boron decoration of g−CN is currently gaining attention as this approach shows active influences on the structural and optical properties [111]. The strong van der Waals force attractions between sp^2^ carbon atoms make g−CN poorly dispersible in an aqueous solution, and the strong hydrophilicity is key to ensuring the full development of g−CN-based materials in an aqueous medium. A structure of hydrophilic and high surface area assembled by two-dimensional B-doped g−CN has been successfully prepared to effectively control the band gap and charge separation (Figure 10a–c). The electron density difference (EDD) map and work function results show that B-doped g−CN has a higher distribution of electron density (Figure 10d–g), and a larger work function makes a higher degree of band bending, leading to the rapid transfer of electrons, and thus improving the PEC capability (Figure 10h,i) [112].

**Figure 11:**
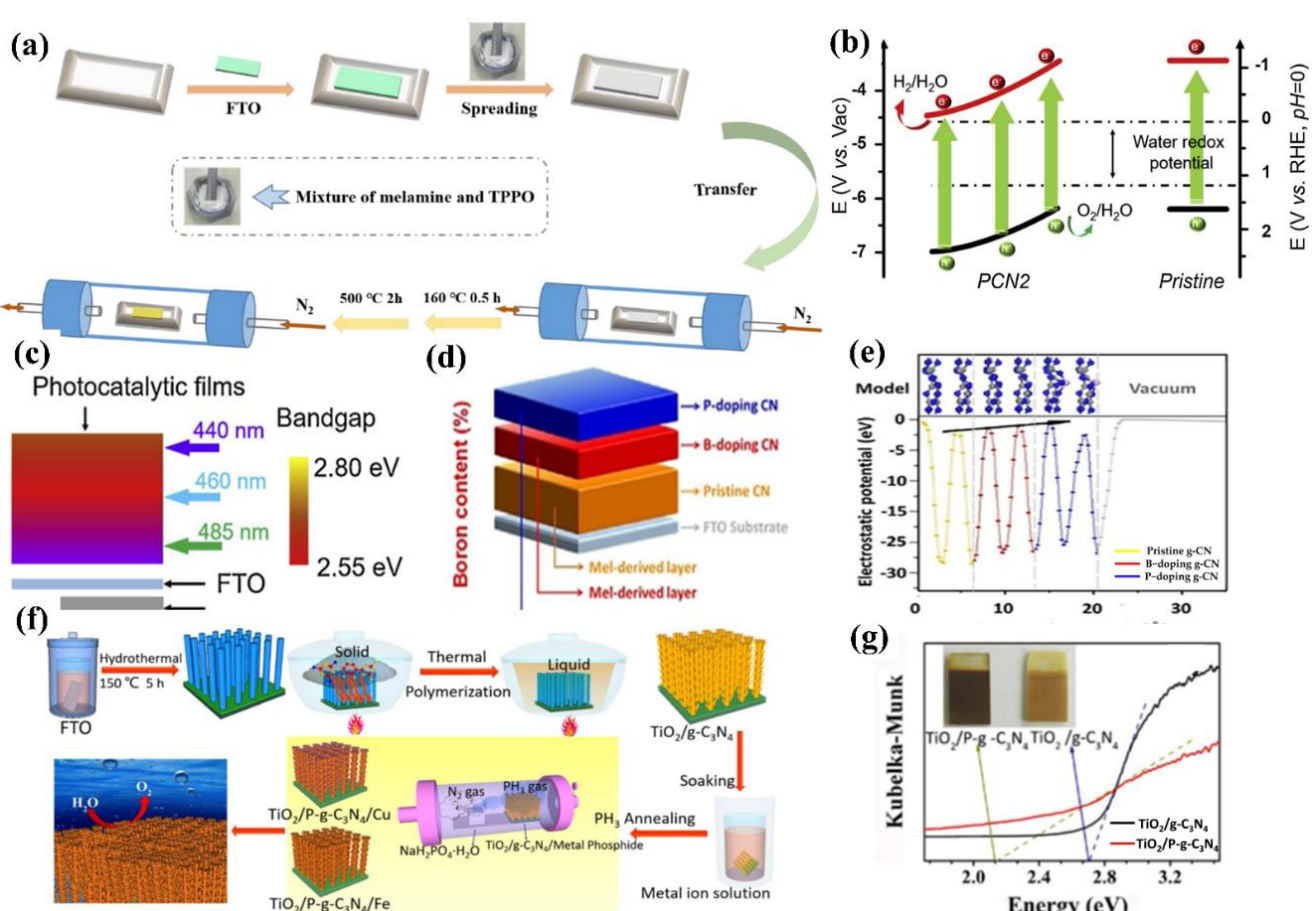
Illustration of (**a**) preparation process of P−doped g−CN photoelectrodes. Reprinted with permission from ref. [113] Copyright 2022 Elsevier. (**b**) A schematic illustration of band structures of gradient S−doped g−CN and pristine g−CN for solar water oxidation, and (**c**) the requirements of the band gap from the top to the bottom of photocatalytic films to allow fully absorbing of solar energy. Reprinted with permission from ref. [116] Copyright 2020 Elsevier. (**d**) Schematic illustration of layer−doped g−CN photoanode composed of ternary layers (P−doped top layer, B−doped middle layer, and pure g−CN bottom layer) and (**e**) electrostatic potential along the c-axis in different doping layers and related models. Reprinted with permission from ref. [122] Copyright 2020 Wiley−VCH. (**f**) A diagram illustrating the fabrication of FTO/TiO_2_−nanorod/P−g−CN/metal phosphide electrodes for the PEC water splitting and (**g**) the Tauc plots. Reprinted with permission from ref. [123] Copyright 2019 Wiley−VCH.

Similarly to B, other non-metallic atoms such as C, P, and S were also examined. Bian and co-workers [53] fabricated a series of C-doped g−CN films by altering the weight percentage of 2, 6−Diaminopyridine and melamine precursors. The embedded gaseous sp^2^ C groups influenced the energy band structure (absorption edge) and improved the diffusion length, resulting in the enhancement of PEC activity. The photocurrent density of C-doped g−CN film (5 wt %) was 0.1 mA cm^−2^ at 1.23 V versus RHE, which was over four-fold higher than that of pure g−CN. Dang et al. [113] prepared P-doped g−CN by utilizing melamine as the g−CN precursor and triphenylphosphine oxide as the P source through a facile solid thermal melting method (Figure 11a). The optimum efficiency was achieved as 127.4 μA cm^−2^, which was five-fold higher than that of pure g−CN. Similar methods were also prepared for the p-doped g−CN with enhanced PEC activity [114].

Other non-metallic atom-doped g−CN photoelectrodes have also attracted extensive attention in PEC applications. Ye et al., 2016 [115], grew a uniform and reproducible g−CN thin film on indium-tin oxide (ITO) substrates by incorporating quantitative thiourea into melamine through a CVD method with a slow heating rate. The S-doped g−CN films displayed a photocurrent of approximately 30 μA cm ^−2^, which was mainly ascribed to the S-doping and particularly the modification of surface morphology, crystalline, and band structure by the polymerization of the precursors.

In addition, Fang et al. [116] modified the g−CN by the incorporation of gradient sulfur doping along the films through molten mediated polymerization of melamine and ammonium thiocyanate. As we know, the band edges have an important role in the thermodynamics of the redox reactions. An ideal design of the photoanodic film and the corresponding charge separation and transfer after the visible absorption are shown in Figure 11b. The valence band maximum of the film should gradually increase from the bottom (close to the substrate) to the top (away from the substrate) versus vacuum potential (vs. Vac), while the conduction band minimum of the film should progressively decrease from the top to bottom (vs. Vac). Therefore, the photo-excited electron will transfer to the counter electrode for hydrogen evolution on the other side. Furthermore, the band-gap of the film should increase from bottom to top. Under illumination, the top of the photoanode will absorb the solar light with a low wavelength. Subsequently, the incident illumination with a higher wavelength will be captured by the bottom active film. As expected, the S-doped g−CN narrowed the band gap from 2.8 to 2.55 eV with a forceful absorption tail in the visible light region. In the depth study, charge separation and migration are enhanced via virtue of the inch-by−inch varied band structures, which makes an agreement with the theoretical example as shown in Figure 11c. As a result, the optimal photocurrent density is ca. 110 μA/cm^2^ at 1.23 V vs. reversible hydrogen electrode. This manifestation acts as a platform to form the gradual variation of the band-gap employing metal-free films to enhance the performance of PEC for other extended applications [117,118,119,120].

Lately, g−CN film via co-doping of nonmetal and metal atoms has gained wide attention because this strategy shows a positive impact on the structural and optical properties. Jing et al. [121] reported the preparation of K and I co-doped g−CN with the use of dicyandiamide, KI, and I_2_ as the starting materials. It was found that the K&I co-doping transforms the from amphoteric to n-type semiconductor property. The photocurrent density of K&I co-doped g−CN is approximately 50.3 μA cm^−2^ at 0.5 V versus Ag/AgCl. Luan et al. [122] reported a unique multi-layer doped non-metal g−CN photoanode using a one-pot TVC. In this structure, a P-doped top layer, B-doped middle layer, and pure g−CN bottom layer were designed to enhance the photocurrent density by nearly nine times that of the pure g−CN. This approach effectively forms dual junctions composed of ternary layers to optimize the charge carrier behavior. The calculated electrostatic potential along the c-axis clearly exhibits that the work function gradually decreases from pure g−CN to B-doped g−CN and P-doped g−CN layers, which is conducive to transfer carrier from bulk to surface (Figure 11d,e).

Based on the discussion above, doping additional atoms and impurities into the g−CN matrix was mainly focused on solid or liquid state reactions. A gas-phase reaction using phosphide as a P source was also achieved by Qin et al. [123]. Clearly, a gas reagent can diffuse into the interlayer and produce a more homogeneous modification. Moreover, a simple phosphide gas treatment of TiO_2_ nanorod/g−CN not only narrows the band gap into a visible-light range from 2.70 to 2.13 eV but also forms a metal (Cu and Fe) phosphide water oxidation cocatalyst via one-step phosphide annealing process. As a result, the metal phosphide-modified sample yields an outstanding visible light (>420 nm) photocurrent response of approximately 0.3 mA cm^−2^ at 1.23 V versus RHE (Figure 11f,g). This strategy will provide new insight into other correlative and highly applicable 2D semiconducting materials.

In fact, heteroatom doping is deemed to introduce impurities into g−CN films, with the doping atoms either substituting lattice atoms or dwelling in the in-planar cave of g−CN. In addition, the incorporation of dopant orbitals into the g−CN molecular orbitals enables us to modulate the light absorption and redox band potentials. The above-discussed investigations on the novel doping approach can be further applied to other modified g−CN-based films, which hold immense potential for other applications such as organic pollutants degradation and CO_2_ reduction. However, excessive doping of heteroatom is detrimental to PEC efficiency because the appearance of more defects reorganizes the light-generated electron/hole pairs due to the doping asymmetry [119,124,125].

### 5.2. Copolymerization of g−CN

The π−conjugated structure of g−CN is another factor that influences the PEC activity owing to the sp^2^−hybridization of carbon and nitrogen elements. The copolymerization approach allows the integration of structure-matching aromatic groups or organic additives into the g−CN framework to adjust its intrinsic properties [126,127,128]. It was found that molecular doping can modulate the conventional π systems, band structure, electronic properties, optical absorption, and, importantly, PEC performance [129]. Bian et al. [53] copolymerized melamine and 2,6−diaminopyridine (26D) to synthesize g−CN film by the TVC method. The XPS demonstrates that the 26D generates gaseous sp^2^ C groups under high temperatures and increases the C/N ratio of g−CN film to promote electron transport. The resulting film shows strong light response properties as well as an extended light absorption region, resulting in the improved photocurrent density from approximately 0.024 to 0.1 mA cm^−2^ at 1.23 V versus RHE (nearly four times).

**Figure 12:**
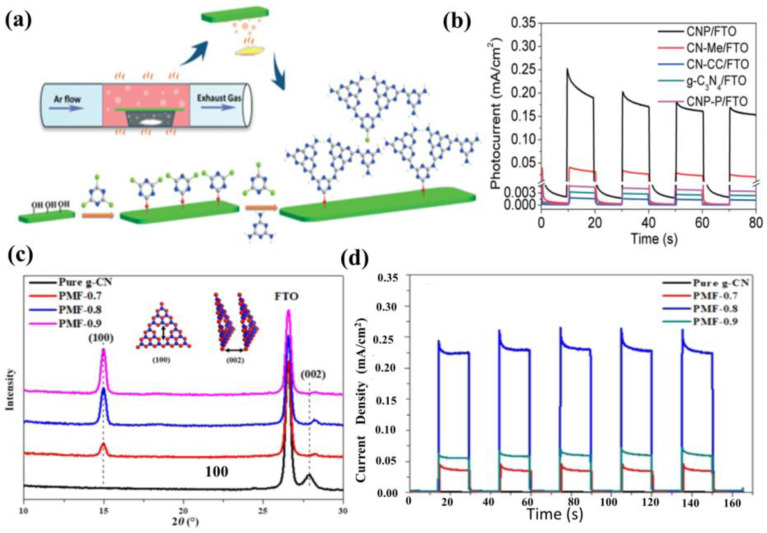
(**a**) Schematic illustration of the synthesis of the g−CN film with a triazine–heptazine network, and (**b**) photocurrent (i–t curve) of g−CN film chopping in 0.5 M Na_2_SO_4_ under simulated solar irradiation. Reprinted with permission from ref. [130] Copyright 2019 Royal Society of Chemistry. (**c**) XRD patterns of pure g−CN and tailored films, and (**d**) transient photocurrent response in 0.2 M Na_2_SO_4_ at 1.23 V vs. RHE. Reprinted with permission from ref. [131] Copyright 2018 Wiley-VCH.

In another work, Jia et al. [130] prepared a compact 2D carbon nitride polymer film (CNP) on FTO through an evaporation polymerization strategy, utilizing melamine and cyanuric chloride as raw materials. The photocurrent density of the CNP film is 230 μA cm^−2^ at 1.23 V (vs. RHE) under visible light irradiation without sacrificial reagents and cocatalysts, which is 7.5, 176.9 and 67.6 times higher than that of the CN-Me (melamine as a precursor), CN-CC (cyanuric chloride as a precursor), and bulk g−CN powders, respectively. The improved PEC performance is ascribed to the enhanced charge transport, increased number of terminal amino groups, and the efficient visible light harvesting caused by the intimate contact (Figure 12a,b). According to the report by Xiong et al. [131], a synergistic protocol is employed to combine the formaldehyde modification and sulfur doping methods to tune (100) and (001) crystal facets of g−CN. This strategy yielded tremendous enhancement in photocurrent response, reaching nearly 228.2 μA cm^−2^ at 1.23 V vs. RHE (Figure 12c,d).

**Figure 13:**
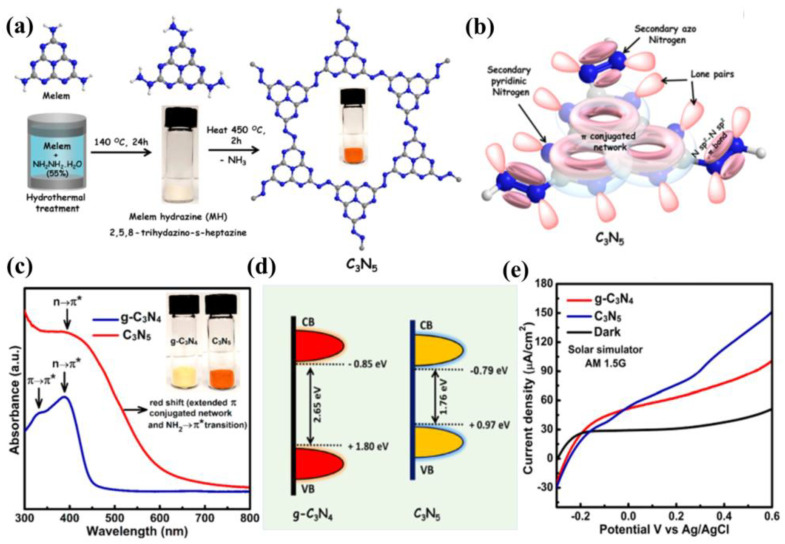
(**a**) Synthesis strategy of C_3_N_5_ from melem by melem hydrazine. Gray, blue, and white balls denote C, N, and H, respectively. (**b**) Plausible molecular orbital overlap schematic diagram of C_3_N_5_. (**c**) DR−UV-vis absorption spectra (the inset shows the photographs of g−C_3_N_4_ and C_3_N_5_ samples). (**d**) Density of state (DOS) revealing band-gap structure of g−C_3_N_4_ and C_3_N_5_. (**e**) LSV photocurrent response of C_3_N_5_ and g−C_3_N_4_ blended TiO_2_ sample measured in 0.1 M Na_2_SO_4_ solution AM1.5G light irradiation and under dark conditions. Reprinted with permission from ref. [132] Copyright 2019 American Chemical Society.

**Figure 14:**
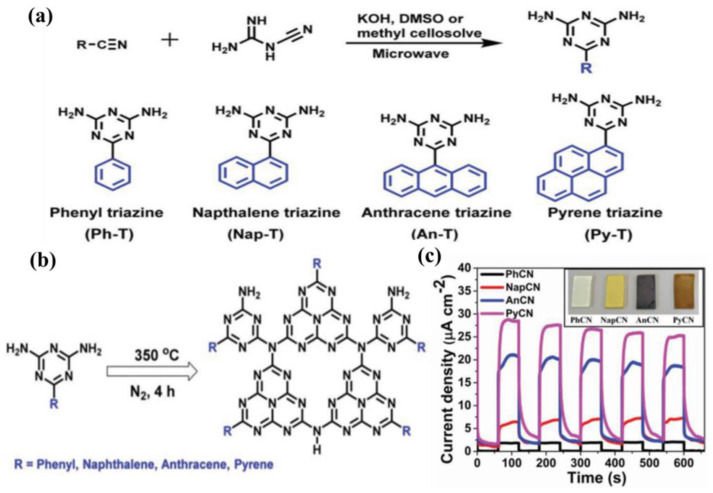
(**a**) Synthesis diagram of polycyclic aromatic triazines and their different molecular structures. (**b**) Self-condensation of the corresponding monomers. (**c**) The photocurrent measurements of polycyclic aromatic hydrocarbons modified CNs (PAH-CN) drop-casted electrodes at 1.23 V vs. RHE (the inset shows the photographs of g−CN dissolved in tetrahydrofuran (THF) and drop-casted on FTO). Reprinted with permission from ref. [133] Copyright 2019 Wiley-Blackwell.

In view of the advantages of chemical structure modification and copolymerization, Kumar et al. [132] synthesized C_3_N_5_ polymer by applying melamine-derived melem (2,5,8−triamino-s−heptazine) and inorganic monomer (hydrazine hydrate) to generate intermediate melem hydrazine (MH) through hydrothermal reaction, following annealing at 450 °C for 2 h. In the C_3_N_5_ framework, two s-heptazine units are bridged together by azo linkage (−N≡N−), which is a completely different bonding mode compared to C_3_N_4,_ where three heptazine units are connected with tertiary nitrogen. Owing to overlap between the π orbitals of azo-bridged units and the π system of heptazine motif, the band-gap of C_3_N_5_ was markedly reduced to 1.76 eV, which enables the optical light absorption to expand up to 670 nm. The photocurrent response for the C_3_N_5_ and g−C_3_N_4_ blended TiO_2_ electrode was proved to be 152 and 100 μA cm^−2^ at 1.23 V vs. NHE (Figure 13a–e). Recently, a high dispersible carbon nitride material functionalized with polycyclic aromatic hydrocarbons (PAH-CNs) was first introduced by Karjule et al. [133]. New starting monomers with substituted triazines were fabricated through a microwave (MW) irradiation strategy using dicyandiamide and aryl-nitrile as raw materials, which forms PAH-CNs by thermal condensation at 350 °C. Furthermore, changing the PAH group within the g−CN scaffold enables the modulation of their optical and electronic properties. The photocurrent density is improved from approximately 2 to 27 µA cm^−2^ (nearly 13 times) owing to the more extended conjugation to promote electron transport and expand light-harvesting range (Figure 14a–c). This breakthrough work can permit researchers to fabricate well-dispersed g−CN materials with tunable electronic and improved light-harvesting properties for PEC or other applications.

To sum up, doping is used to modulate the π conjugated system including light absorption, electron band structure-properties, and PEC activity by introducing heteroatoms, aromatic groups, or organic additives. It can be divided into atomic doping and molecular doping according to the doping levels. In general, atomic doping involves the insertion of non-metallic atoms into the g−CN lattice, partially replacing C or N atoms. On the other hand, the metal atoms can be inserted into the triangular gap cavity of the g−CN lattice. Compared with metal doping, non-metallic doping has obvious advantages because it can retain the non-metallic properties of g−CN. Co-doping and gradient doping have also attracted extensive attention. However, excessive heteroatomic doping will lead to doping asymmetry and a large number of defects, which will lead to the recombination of photo-generated carriers, which will hinder the improvement of PEC efficiency. Copolymerization is a molecular doping method in which the addition of structure-matching organic reagents (such as organic comonomers containing cyanide and benzene rings) in the copolymerization of nitrogen-rich precursors is an effective method to adjust their band structure at the molecular level of g−CN.

### 5.3. Cocatalysts

**Figure 15:**
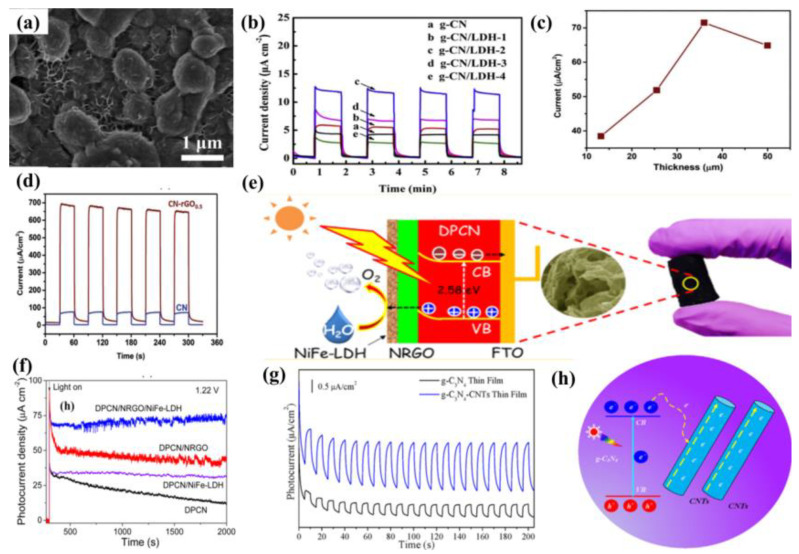
(**a**) SEM images of g−CN/LDH with 10 mC electric quantity. (**b**) i–t curves of g−CN and g−CN/LDH composites with different LDH amount under chopped light. Reprinted with permission from ref. [141] Copyright 2019 Elsevier. (**c**) The photocurrent-thickness relationship. (**d**) Photocurrent response in 0.1M KOH and10% TEOA aqueous solution at 1.23 V vs. RHE upon illumination of one sun. Reprinted with permission from ref. [145] Copyright 2018 Wiley-VCH. (**e**) The photographs, FESEM image and charge transfer mechanism. (**f**) Transient photocurrent responses under AM 1.5G irradiation at 1.22 V vs. RHE. Reprinted with permission from ref. [142] Copyright 2016 American Chemical Society. (**g**) Photocurrent response of g−CN−CNTs compared with pure g−CN thin film in 0.5M Na_2_SO_4_ at 0 V vs. RHE. (**h**) Schematic illustration of charge transfer mechanism. Reprinted with permission from ref. [91] Copyright 2017 Elsevier.

The modification of semiconductors with a cocatalyst is also a fascinating endeavor to enhance PEC performance. Cocatalysts mainly play two important roles, namely, acting as a reactive site and facilitating the separation of photo-induced charge. The typical reduction cocatalysts employed in the g−CN photoelectrodes are noble metals such as Pt, Rh, Ru, and Au. Nevertheless, it is not practical for commercial g−CN-based PEC applications owing to their high cost and rarity. In terms of economic and practical production, much research has been conducted on the use of transition metals (such as Ni [13] and Cu [134]) or alloying Pt with less expensive and abundant metals as co-catalysts. For example, nickel-decorated sulfur-doped graphitic carbon nitride with bismuth oxybromide (Ni/S-g−CN/BiOBr) shows a photocurrent density of 177.2 uA/cm^2^ at 1.23 V vs. RHE, which is nearly three-fold higher than that of S-g−CN/BiOBr [13]. In addition, bimetallic co-catalysts have a great advantage in promoting the PEC reaction in comparison with monometallic analogues. The excited electrons from semiconductors can be injected into bimetallic alloys more easily. The bimetallic Pt-Ni incorporated g−CN nanotubes exhibit an excellent and stable photocatalytic performance, which is higher than that of Pt/g−CN nanotubes [135]. This result shows that bimetallic is expected to replace noble metal with better performance and lower cost. Furthermore, hydroxides [136] and oxyhydroxides [137,138] are effective cocatalysts for the g−CN modification to improve the redox reactions activity [139,140]. The photocurrent density could be availably improved from 89 μA cm^−2^ to 0.12 mA cm^−2^ after loading FeOOH on the g−CN film surface [51]. In another work, Fan et al. [141] modified g−CN film with NiCo-layered double hydroxide (NiCo-LDH) co-catalysts via cathodic electrochemical deposition. The resulting photoelectrode after incorporation of the NiCo-LDH decreases the charge migration resistance at the interfaces, which helps to promote the reaction kinetics and charge separation. The optimized g−CN/NiCo-LDH photoelectrode shows a photocurrent up to 11.8 μA cm^−2^ at 0.6 V vs. SCE, which was 2.8−fold higher than that of pristine g−CN (Figure 15a,b).

Because of the superior electrical conductivity of sp^2^−bonded carbon atom, carbon-based materials, e.g., carbon dots, carbon nanotubes (CNT), and graphene oxide, etc., are promising substrates and excellent reductive cocatalyst to integrate with g−CN, inhibiting the recombination of charge carriers [142,143,144]. Graphene, a honeycomb nanostructured 2D macromolecular structure of carbon atoms, has received tremendous attention because of its excellent optical and electrical features, large surface area, and good chemical stability. There are growing numbers of research on the fabrication of graphene/g−CN hybrid photoelectrodes for PEC applications by considering 2D/2D face-to−face interaction. In one of the pioneer studies [145], a hybrid material with the carbon nitride layer on the reduced graphene oxide layer (CN-rGO) was synthesized by a doctor-blade technique using a supramolecular precursor followed by thermal calcination. The incorporated graphene behaves as the electron-conducting channel to improve both photo-generated charge separation and electron diffusion length. With increasing graphene contents, the hybrid material exhibited a higher photocurrent response of 72 µA cm^−2^ at 1.23 V versus RHE (electrode thickness up to 36 µm) and subsequently decreased for a thicker photoelectrode. Photocurrent measurements with holes scavenger (TEOA) display a photocurrent response of 660 µA cm^−2^ at 1.23 V versus RHE and the external quantum yield of 60% at 400 nm (Figure 15c,d).

Recently, graphene derivatives (e.g., chemically doped graphene) have been integrated with g−CN to form a heterojunction photoelectrode. In particular, various types of dopants have triggered keen interest in the preparation of N-doped graphene as it can ameliorate the optical, electronic, and chemical reactivity of graphene by making use of the lone-pair electrons of nitrogen atoms and the π−conjugated electrons of 2D graphene. Hou and coworkers [142] employed a facile hydrothermal method with urea to integrate N-deficient porous g−CN nanosheets (DPCN) and Nickel-iron−layered double hydroxide (NiFe-LDH) into 3D N-doped graphene (NRGO) framework. A record-high photocurrent density of 162.3 μA cm^−2^ at 1.4 V and remarkable photostability (>10 h) are realized (Figure 15e,f). The enhancement in the efficiency of this novel aerogel photoanode is based on: (i) the optimized energy band structure and improved light absorption derived from the 2D DPCN with deficient structures, (ii) 3D NRGO-based aerogel provides a hierarchical porous structure to improve the charge transport property, and (iii) a close-contacting NiFe-LDH contributes to the effective kinetic transportation of photo-generated electron–hole pairs via introducing the spatially separated interfaces.

In addition, 1D CNT is another carbon allotrope with extraordinary electronic properties. Hitherto, there have been several investigations to integrate g−CN with CNT as the composite films for promising PEC applications. Miao et al. [91] employed a vacuum filtration self-assembled process and subsequent annealing treatment to synthesize g−CN−CNTs thin film by incorporating g−CN nanosheets supernatant with CNTs aqueous solution. The photocurrent density could be availably improved from approximately 0.5 to 1.8 μA/cm^2^ (nearly 3.7 times) via the recombination of CNTs to promote electron transport. The enhanced PEC performance was accredited to the excellent electrons transport capability by CNTs, fundamentally resolves the poor electronic conductivity of the conglomerated g−CN nanosheets structure and allows valid electrons separation and collection (Figure 15g,h).

Among the surface modification methods to improve the PEC performance of g−CN, the supported co-catalyst is an effective one to improve the separation efficiency of photo-generated carriers. The co-catalyst can not only promote the activity of the substrate semiconductor but also inhibit the photo-corrosion that may occur during the PEC process. However, non-noble metal-based co-catalysts still encounter low activity and low stability. In addition, the energy level structure, electronic structure and interface structure also require reasonable construction for the g−CN/co-catalyst system. There are many factors affecting the PEC performance of the g−CN/cocatalyst system, including load, particle size, interface binding force, etc. Only after reasonable consideration and design can the photo-generated carriers be efficiently generated from g−CN and across the contact interface to the cocatalyst surface in the right direction. In addition, a photocurrent density comparison of the functionalization of g−CN-based photoelectrodes by heteroatoms doping and textural design is shown in Table 3.

## 6. Composite Films of g−CN as Highly Valid Photoelectrodes for PEC Application

As aforementioned, similar to the conventional inorganic semiconductor photocatalysts, the pristine g−CN films usually suffer from several intrinsic shortcomings, such as insufficient visible-light absorption, the rapid recombination rate of charge carriers, and low electrical conductivity [105,146,147]. Therefore, various strategies are employed to restrain the recombination of charge carriers in g−CN films to enhance the PEC performance. In addition to the heteroatoms doping and textural design, as discussed in Section 5, the construction of g−CN-based heterojunction composite films for PEC application has elicited a great deal of attention, which will be across-the−board reviewed in the following subsections. 

**Figure 16:**
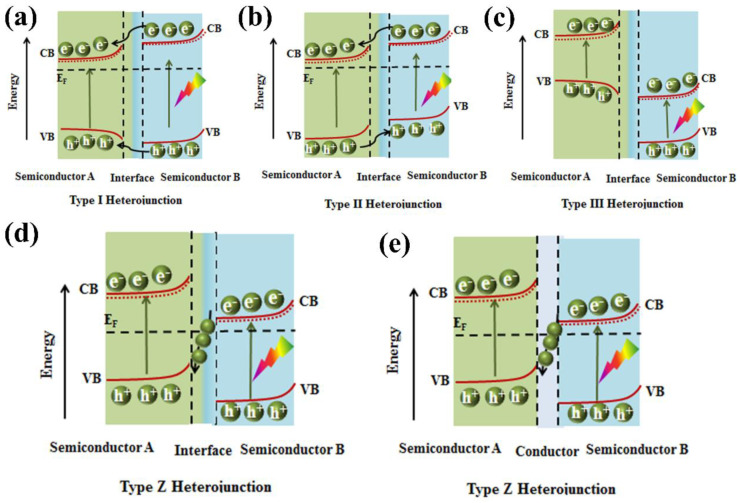
Schematic illustration of the transfer of electron-hole pairs for various types of heterojunction composite films: (**a**) Type I, (**b**) Type II, (**c**) Type III, (**d**) semiconductor-semiconductor Z-scheme, and (**e**) semiconductor-conductor−semiconductor Z-scheme.

Typically, a g−CN-based heterojunction photoelectrode is engineered by combining two different semiconductors with appropriate valence band (VB) and conduction band (CB) potentials, which results in a new electronic structure after thermal equilibration. In other words, band bending forms at the heterojunction interface on account of potential differences between two sides of composite films [148]. This transformation induces a built-in electric field to spatially separate and transfer the photo-generated electrons and holes. Subjected to the separation mechanism of charge carriers and VB and CB potentials of semiconductors, the g−CN-based composites can be categorized into four classifications, i.e., Type I, Type II, Type III, and Z-scheme (Figure 16) [3]. For the Type I heterojunction (Figure 16a), both the VB and CB potentials of semiconductor B with a smaller band gap are within the larger band-gap (semiconductor A), creating a straddling band alignment. The band edge positions of Type I heterojunction lead to the transfer of charge carriers in a single direction. Since all charges are accumulated in a semiconductor B, this will result in the commonly impaired photoredox efficiency. For the Type II heterojunction, the band edge potentials are staggered between both semiconductors (Figure 16b). As a result, the photo-generated electrons migrate to a less negative CB potential of semiconductor A, while the holes migrate to a less positive VB potential of semiconductor B. This significantly improves the spatial separation and migration of carriers to restrain the charge recombination [149,150]. However, the enhancive separation efficiency can be obtained based on the Type II heterojunction. The main drawback was caused by the chemical potential between both semiconductors, leading to a decrease in oxidation and reduction capacity. Recently, a new Z-scheme heterojunction was reported to surmount this shortfall. According to the electron mediator, the g−CN-based Z-scheme mechanism can be categorized into two typical forms: the semiconductor-semiconductor Z-scheme heterojunction (Figure 16d) and semiconductor-conductor−semiconductor Z-scheme heterojunction (Figure 16e). The solid electron mediator includes reduced graphene oxide [151,152] and noble-metal nanoparticles such as Au [153,154], Ag [155,156,157] and Cd [158], etc. For the former [159,160,161], the electron in the CB of semiconductor B directly transfers to the VB of semiconductor A, leading to the valid separation of the electrons in the CB of semiconductor A and the holes in semiconductor B. While for the latter, a conductor acts as an electron mediator and can facilitate the electron migration from semiconductor B to semiconductor A [162]. Early studies also demonstrated that interface engineering by introducing an electron mediator is beneficial to improving the photocatalytic performance of the Z-scheme heterojunction compared to the direct Z-scheme one [163,164]. In the type III heterojunction (Figure 16c), the VB and CB of semiconductors A and B do not overlap with each other [165].

Based on the above discussions, Type II and Z-scheme pathways that effectively separate the electron-hole pairs are ideal in the preparation of the g−CN-based film. In view of these two types of heterojunctions, selecting an appropriate semiconductor material to couple with g−CN is of utmost significance not only to enhance the optical light absorption but also to facilitate the charge migration and separation to suppress the recombination rate. In the following section, we will critically exemplify recently published studies on these two types of g−CN-based composite films for PEC water splitting.

### 6.1. Type II Heterojunctions

The flexibility, as well as their 2D flat morphology, favors g−CN films as an accessible platform for the construction of heterojunction with various components. Based on the reported literature, a series of categories of g−CN-based heterojunctions have been successfully fabricated: (i) metal oxides/g−CN heterojunction, (ii) metal sulfides/g−CN heterojunction, and (iii) complex compound/g−CN heterojunction. The detailed studies for g−CN-based heterojunctions, efficiency, and applications will be discussed.

#### 6.1.1. Metal Oxides/g−CN Hybrid Heterostructures Films

Until now, various metal oxide semiconductors have been coupled with g−CN to form type II heterojunction for PEC applications. Among those metal oxides, TiO_2_ is one of the most suitable candidates, and the triumphant formation of TiO_2_/g−CN heterojunctions has been reported to improve the PEC activity.

**Figure 17:**
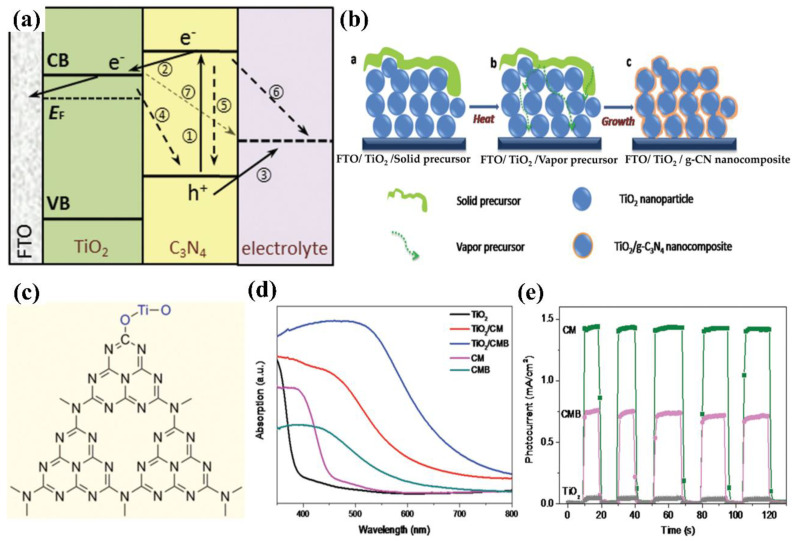
(**a**) Schematic illustration of charge transfer mechanism of type (II) TiO_2_/g−CN photoelectrode (paths 1, 2, and 3, charge excitation and transfer; paths 4, 5, 6, and 7, charge recombination). Reprinted with permission from ref. [166] Copyright 2017 John Wiley and Sons Ltd. (**b**) Schematic illustration of the synthesis of the TiO_2_/g−CN thin film. (**c**) Schematic chemical structure of TiO_2_/g−CN. (**d**) UV–vis absorption spectra of the TiO_2_, TiO_2_/CM, TiO_2_/CMB substrates, and g−CN powders made from CM (cyanuric acid-melamine) and CMB (cyanuric acid-melamine−barbituric acid). (**e**) Photocurrent response in 0.1 M Na_2_S under white light (λ > 410 nm). Reprinted with permission from ref. [168] Copyright 2015 John Wiley and Sons Ltd.

Albero et al. [166] analyzed the electron transfer kinetics between g−CN and mesoporous TiO_2_ using laser transient absorption spectroscopy (L-TAS) (path 2, Figure 17a), suggesting the effective separation of the electron. However, the ineffective diffusion of the hole to the electrolyte (path 3, Figure 17a) was found to be the restricting process during the PEC performance. Enlightened by this conception, combining TiO_2_ with g−CN is the most feasible strategy to achieve a large-scale g−CN-based heterojunction photoelectrode with high PEC efficiency. Sima et al. [167] fabricated a mesoporous TiO_2_/g−CN hybrid nanostructure film, in which exfoliated g−CN were immobilized on mesoporous TiO_2_ via a spray coating approach. In this architecture, the g−CN exposes its edges to the visible light and dispersion liquid that facilitates fast electrons and holes transport. The prepared TiO_2_/g−CN hybrid film possessed a high photocurrent density of 0.32 mA cm^−2^, which was higher than those of pristine g−CN and pure TiO_2_ films. Inspired by the aforementioned approach, Xu and co-workers [168] coated g−CN on the mesoporous TiO_2_ through an in situ vapor-transport growth approach by calcining a supramolecular mixture of cyanuric acid-melamine (CM) precursor. As expected, its photocurrent reached 1.4 mA cm^−2^, which was a 30−fold enhancement compared to pristine TiO_2_ (0.045 mA cm^−2^). They also studied the impact of barbituric acid molecules on the mixture precursor on the performance of g−CN/TiO_2_. A relatively high carbon doping content augments the defect levels and causes higher recombination of charge carriers, resulting in a decreased photocurrent to 0.7 mA cm^−2^.

The improved PEC efficiency was in virtue of the prominent interface-bound energy transition of g−CN/TiO_2_. In addition, the direct Ti–O–C bond creates supererogatory molecular orbitals at the interface, leading to a strong red-shift of the optical visible-light absorption (Figure 17b–e). The contribution of TiO_2_/g−CN photoelectrodes is obviously affected by their shape, size, and nanostructures of TiO_2_. Among various nanostructures, one-dimensional (1−D) TiO_2_ nanostructures such as nanowires and nanotube arrays have drawn comprehensive interest owing to their high specific surface area and pore volume [169,170,171,172]. Sun et al. [173] reported the synthesis of g−CN/TiO_2_ nanotube array heterojunction by the vapor-pyrolysis of anodized Ti sheets over melamine. The obtained g−CN/TiO_2_ nanotube heterojunction exhibited strong optical absorption and reduced impedance compared to the pure TiO_2_ nanotube. In addition, it also demonstrated enhanced PEC activity and stability. In another work, Liu and co-workers [174] reported g−CN/TiO_2_ nanotube array (TNTAs) heterojunction with a g−CN shell and TNTAs core through TVC employing melamine and cyanuric acid supramolecular complex as the precursors. A noticeably enhanced photocurrent density of 0.86 mA cm^−2^ was realized, which was 4.5−fold and 2−fold higher than those of g−CN/amorphous TiO_2_ heterojunction and pure TiO_2_ nanotubes. It is worth emphasizing that the crystalline TiO_2_ nanotubes offer a more distinct photo-response due to their unique one-dimensional nanostructure in comparison with the amorphous TiO_2_ film.

**Figure 18:**
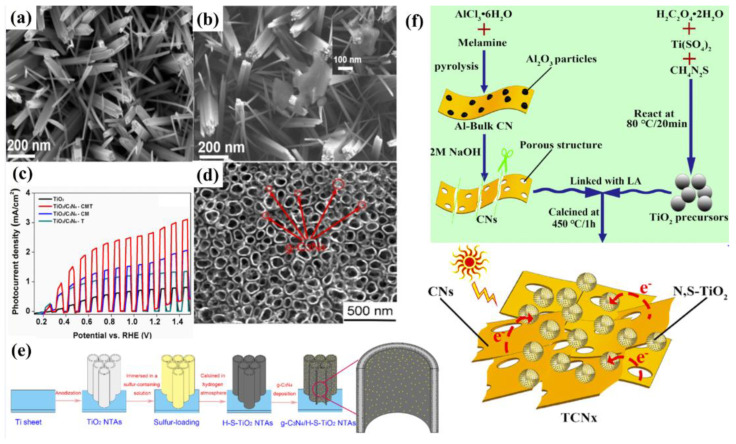
FE-SEM images of (**a**) pure TiO_2_, (**b**) TiO_2_/g−CN−CMT. (**c**) LSV photocurrent density in 1MNaOH at 1.23 V vs. RHE. Reprinted with permission from ref. [175] Copyright 2020 Elsevier. Top-view SEM images of (**d**) g−CN/H-S−TiO_2_ NTAs4. (**e**) Schematic illustration of fabrication progress. Reprinted with permission from ref. [178] Copyright 2020 American Chemical Society. (**f**) Schematic illustration of the preparation. Reprinted with permission from ref. [179] Copyright 2021 Elsevier.

In addition, Bhat et al. [175] reported the combination of g−CN and TiO_2_ nanorods using a dip-coating process with cyanuric acid-melamine–thiourea (CMT) complexes as starting materials. Using CMT complexes for the fabrication of TiO_2_/g−CN heterojunction is more advantageous than the individual materials. The photocurrent density can reach up to 2.74 mA cm^−2^ at 1.23 V (vs. RHE) (Figure 18a–c). In another work, Su and co-workers [176] synthesized modified vertically aligned TiO_2_ NTs with P-doped g−CN via anodization and wet-dipping followed by post-annealing. 1−butyl−3−methylimidazolium hexafluorophosphate (BmimPF_6_) was added as a cheap phosphorus source. P-CN/TiO_2_ NTs exhibited the photocurrent response (1.98 mA cm^−2^ at 0 V vs. Ag/AgCl), which was higher than that of pristine TiO_2_ NTs. This promotion was because of the synergic mechanism between the introduction of P heteroatoms and the improvement of π−electron delocalization by 2−aminobenzonitrile (ABN). A previous study revealed that hydrogenation can improve the visible-light absorption range, structure matching, and stability [177]. Inspired by this, Zhou et al. [178] reported a g−CN/S-doped hydrogenated TiO_2_ NTAs (g−CN/H-S−TiO_2_ NTAs) photoelectrode using a dip-coating approach. The results exhibited a higher transient photocurrent response of 1.64 mA cm^−2^ with good stability compared to TiO_2_ NTAs (0.29 mA cm^−2^). Hydrogenation reduces the energy gap of TiO_2_ due to the introduction of Ti^3+^ and oxygen vacancies. S-doping further forms a new impurity level that upshifts the TiO_2_ VB edge. The introduction of g−CN can form type II heterojunction with H-S−TiO_2_ NTAs, realizing an effective separation of charge and extended visible-light absorption (Figure 18d,e). Recently, the structure of high surface area assembled by chemical tailoring g−CN has been successfully prepared (Figure 18f). Additionally, combining with N,S co-doped TiO_2_ significantly improves the PEC activity [179]. Improvement of the efficiency of TiO_2_−based photoanodes using nickel-modified polymeric carbon nitride has also been explored [180].

Quantum dot (0D) semiconductors help to widen band structures and enhance redox capability and open-circuit voltage compared with their bulk counterparts (3D) [181,182]. Recently, g−CN quantum dots (CNQDs) have been attracting a lot of attention because of the narrow size distribution, as well as high fluorescence [183]. Su et al. [184] fabricated TiO_2_ nanotube arrays (NTAs) with CNQDs via a delicate organic molecular linkage. The process included immersing TiO_2_NT in CNQDs aqueous solution with 1 v% 3−mercaptopropionic acid (MPA) at 80 °C for 12 h. The resulting products were rinsed and dried in air. The MPA-modified TiO_2_NT/CNQDs achieved a photocurrent response of 1.34 mA cm^−2^ at 0.3 V vs. Ag/AgCl, which was mainly ascribed to the effective heterojunction structure and particularly the strong affinity of TiO_2_ NTAs.

**Figure 19:**
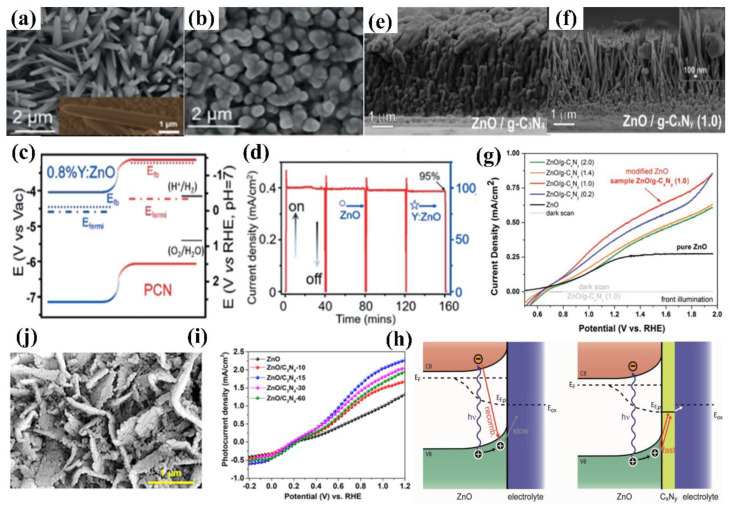
Top−view SEM images of (**a**) 0.80%Y:ZnO NRs, (**b**) 0.8%Y:ZnO/g−CN core-shell photoanode (the inset is the single 0.8%Y:ZnO NR). (**c**) Schematic illustration of the energy band alignment and water redox potentials. (**d**) The stability test of the core-shell photoanode at 1.23 V vs. RHE. Reprinted with permission from ref. [188] Copyright 2018 John Wiley and Sons Ltd. (**e**,**f**) Cross−section FEG−SEM images of (**e**) ZnO nanowires (NWs) coated with cyanuric acid−melamine (CM) complex as the source of g−CN followed by using powder deposition technique, and (**f**) ZnO-NWs treated with CM complex followed by using dip−coating deposition. (**g**) LSV measurements of the samples fabricated via dip−coating different concentrations of CM dispersion. (**h**) Schematic of the proposed working mechanism. Reprinted with permission from ref. [190] Copyright 2017 John Wiley and Sons Ltd. (**i**) LSV curves. (**j**) FESEM images of ZnO/g−CN QDs. Reprinted with permission from ref. [191] Copyright 2020 American Chemical Society.

In addition to the frequently reported TiO_2_/g−CN photoelectrode, ZnO is another pivotal metal oxide with a wide band gap (3.2eV), which can be valid coupled with g−CN [185,186,187]. In one study by Fang et al. [188], g−CN was coated on yttrium-doped zinc oxide Nanorod (Y:ZnO NR) Arrays via in situ thermal vapor polymerization. The prepared Y:ZnO/g−CN heterostructure possessed a photocurrent density of 0.4 mA cm^−2^, over 50−fold that of pure g−CN films achieved by powder. The result reveals the photocurrent retention capability can be preserved at 95% for 160 min. The charge collector layer in type II alignment remarkably reduces the traveling distance of the carrier and minimizes the defect-trapping effect, thereby suppressing the recombination effect (Figure 19a–d). In another similar work, Park et al. [189] loaded the g−CN layer on vertically aligned ZnO nanorods through thermal vapor condensation. In comparison with the individual bare ZnO nanorods or g−CN, the effective charge separation and transportation emerge from the formation of Type II heterostructures. This photoanode demonstrated a photocurrent density of 0.12 mA cm^−2^ and excellent stability up to 5 h.

However, the continuous g−CN layer obtained via in situ thermal vapor polymerization blocks the porous structure. In addition, the as-designed heterostructures also impeded the electrolyte from penetrating into the structure, which may inhibit water oxidation under visible light [168]. Therefore, enhancement of the PEC performance can only be acquired by developing modified synthetic tactics that allows uniform coverage while preserving the porous structure. Hajduk et al. [190] loaded the g−CN layer on the ZnO Nanowires by forming a seed layer of cyanuric acid-melamine (CM) supramolecular complex, followed by their conversion to carbon nitride at high temperature. The successful retaining of the porous structure in the ZnO/g-C_x_N_y_ (1.0) sample was verified by SEM image analysis (Figure 19e–h). The resulting ZnO/g-C_x_N_y_ (1.0) photoelectrode possesses a photocurrent density of approximately 0.25 mA cm^−2^, more than 3.5−fold that of the pure ZnO. Mahala et al. [191] loaded the g−CN QDs on the ZnO 2D nanosheets through a dip-coating method. In comparison with pure ZnO, the ZnO/g−CN QDs heterojunction shifts the optical absorption to higher wavelength ranges with stronger absorbance in the visible light and improves the charge-carrier separation efficiency. The best-obtained ZnO/g−CN photoanode exhibits approximately 2.29 times as high photocurrent density (0.952 mA cm^−2^) compared to bare ZnO at 0.5994 V versus RHE (Figure 19i,j).

Other than the recombination with UV-active TiO_2_ and ZnO semiconductor, WO_3_ has received prodigious attention as a promising candidate owing to its relatively lower band gap (2.5–2.8 eV), which lies in the visible-light range, thus increasing the potential for wider utilize in the PEC applications [192,193,194].

**Figure 20:**
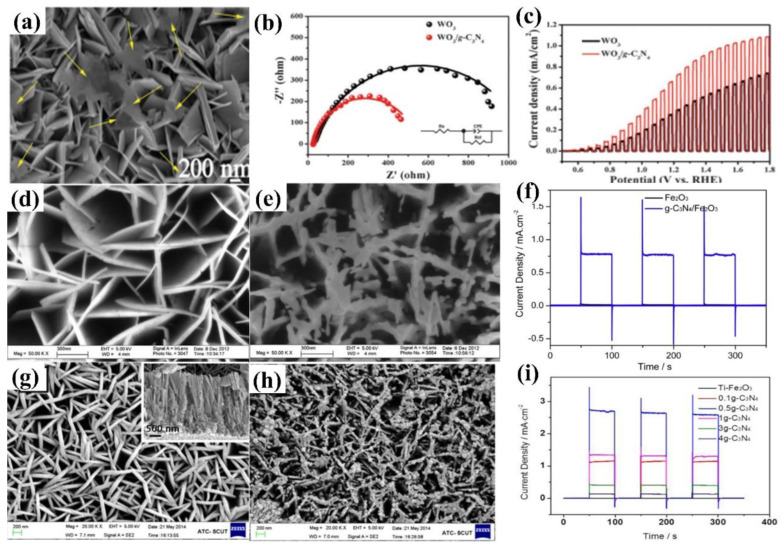
(**a**) SEM image of WO_3_/g−CN NSAs. (**b**) Electrochemical impedance spectra. (**c**) I–V curves measured at a scan rate of 5 mV s^−1^. Reprinted with permission from ref. [195] Copyright 2016 American Chemical Society. Top-view SEM images of (**d**) Fe_2_O_3_, (**e**) g−CN/Fe_2_O_3_. (**f**) I-t curves in 1.0 M NaOH at 0.23 V vs. Ag/AgCl. Reprinted with permission from ref. [197] Copyright 2014 Elsevier. Top-view SEM images of (**g**) Ti−Fe_2_O_3_, (**h**) 0.5g−CN/Ti−Fe_2_O_3_ (the inset is the cross-section of Ti−Fe_2_O_3_). (**i**) I-t curves in 1.0 M NaOH at 0.23 V vs. Ag/AgCl. Reprinted with permission from ref. [199] Copyright 2016 Elsevier.

Li et al. [195] deposited 2D/2D WO_3_/g−CN nanosheet arrays (NSAs) on an FTO substrate through a hydrothermal growth and post-annealing process. It drastically suppressed the recombination of carriers and prolonged the lifetime of electrons manifested by EIS analyses for the WO_3_/g−CN heterojunction. The photocurrent density reached 0.73 mA cm^−2^ at the bias of 1.23 V vs. RHE (Figure 20a–c). Zhan et al. [193] synthesized a g−CN/WO_3_ heterostructure plate array film by a combination of hydrothermal and dipping-annealing strategies. When the content of g−CN was 2.13 at%, the obtained g−CN/WO_3_ heterojunction films exhibited the highest photocurrent density of 2.10 mA cm^−2^ at +2.0 V (vs. RHE), which was three-fold higher than that of pure WO_3_ under illumination. Li et al. [196] uniformly coated g−CN nanosheet on WO_3_ nanorod surface through the dip-coating method, which can achieve efficient visible-light−governed PEC water splitting. The photocurrent response of the g−CN/WO_3_ photoanode achieved 1.92 mA/cm^2^ at 1.23 V vs. RHE. The heterojunction was approximately stable, but distinct degradation (only decreases to 1.85 mA cm^−2^) was observed after 400 min of illumination. The stability performance promotion was realized via dipping WO_3_ nanorod into g−CN nanosheet dispersion for various impregnation times without intermediary drying steps. The enhanced PEC performance is attributed to the synergic effect between g−CN and WO_3_ originating from their well-matched overlapping band structures, which can availably boost the charge separation and the improvement of electrons’ lifetime.

Apart from that, combining g−CN with Fe_2_O_3_ has also recently been studied for PEC application. Liu et al. [197] fabricated g−CN/Fe_2_O_3_ photocatalysts via electrodeposition followed by chemical vapor deposition. By altering the filling degrees of the melamine during the annealing process, the morphology of the g−CN/Fe_2_O_3_ changes from regular nanosheets toward porous cross-linked nanostructures. The photocurrent density of the g−CN/Fe_2_O_3_ is almost 70−fold higher than that of individual Fe_2_O_3_ (Figure 20d–f). The enhancement of PEC efficiency was assigned to the morphology change and the synergy effect of the heterojunction. In previous work, the Ti-doped Fe_2_O_3_ showed an ameliorative conductivity, thus achieving an enhancement in the photocatalytic performance in contrast to bulk Fe_2_O_3_ [198]. Inspired by these facts, Liu et al. [199] established synthetic tactics to construct g−CN−modified Ti-doped Fe_2_O_3_ nanosheets with different percentages, in which g−CN dispersion in water was immobilized on Ti-doped Fe_2_O_3_ nanoarrays via a dip-coating approach. This strategy yielded tremendous enhancement in photocurrent density, reaching approximately 2.55 mA cm^−2^ at 1.23 V vs. RHE (Figure 20g–i). The results were prospectively higher than those for TVC deposition of undoped Fe_2_O_3_.

Apart from that, other endeavors have been dedicated to combining promising candidates with g−CN to construct various heterojunction photoelectrodes. These include SnO_2_/g−CN [73], NiO/g−CN [200], Sn-doped In_2_O_3_/g−CN [201], Cu_2_O/g−CN [202], MoO_3_/g−CN [203], and all of them showed enhanced PEC activity compared to pure g−CN. The construction of metal oxide-based thin films containing 2D structures is also an effective way to improve PEC activity [204,205].

#### 6.1.2. Metal Sulfides/g−CN Hybrid Heterostructures Films

**Figure 21:**
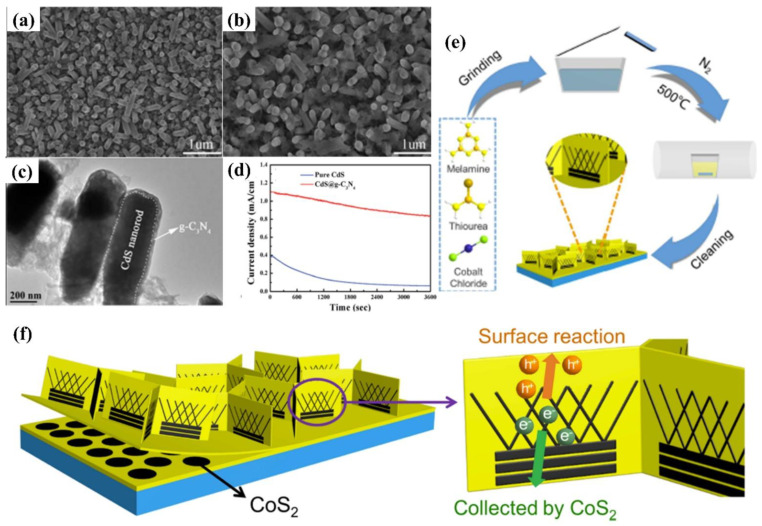
Top-view FESEM images of (**a**) CdS nanorods (NRs) and (**b**) CdS/g−CN core–shell nanorods (CSNRs). (**c**) TEM images of CdS/g−CN core–shell nanorods. (**d**) photostability measurements at 0.9 V versus RHE. Reprinted with permission from ref. [210] Copyright 2015 Royal Society of Chemistry. (**e**) Synthesis procedures. (**f**) The charge transfer mechanism. Reprinted with permission from ref. [206] Copyright 2022 Wiley-VCH.

Along with the wide band-gap metal oxides, the exploitation of g−CN coupled with a narrower band-gap metal sulfide has become a hot research area [206]. CdS, as one of the most indispensable candidates, has attracted comprehensive attention owing to its narrow band gap (~2.4 eV) and great visible-light response (520 nm). In addition, the highly matched energy levels with g−CN are beneficial for the formation of Type II heterostructures, assisting the charge migration and separation under irradiation [207]. In an early study, Li et al. [208] hybridized CdS nanorods (NRs) (5 nm diameters and 20–50 nm lengths) with g−CN nanosheets (NSs) by a facile hydrothermal route and then the dip casting approach. The Cd atoms are embedded steadily in the polymer CN backbone that offers numerous binding sites for CdS nanorods to form intimate contact, resulting in the promoted PEC efficiency. The enhancement was mainly because of increased interfacial contact and a sequential network offered by the long axis of CdS NRs, resulting in effective charge dissociation and transport on both components. On the other hand, CdS seriously suffers from photocorrosion that the sulphide ion (S^2−^) tends to be self-oxidized under strong radiation, and their corresponding photo-generated electron–hole pairs tend to be recombined [209]. In order to improve its stability, protecting CdS from photocorrosion via coating g−CN to form core–shell nanostructures has proven to be an ideal solution. Li et al. [210] synthesized novel nanoarrays composed of CdS/g−CN core–shell nanorods (CSNRs) through a hydrothermal treatment and heating process. The optimal photocurrent density of the obtained CdS/g−CN CSNRs reached up to 1.16 mA cm^−2^, which was approximately 2.5 times that of pure CdS NRs. After 3600 s continuous illumination, there was no obvious loss observed in the photocurrent density (Figure 21a–d).

In another work, Wang and co-workers [211] fabricated an interlocking g−CN/CdS heterojunction film on FTO by electrophoretic deposition and chemical bath deposition strategies. The optimal g−CN/CdS−2h photoanode revealed a maximum photocurrent density of 5.4 mA cm^−2^ at 0.0 V vs. Ag/AgCl under visible light illumination. From their research, what was of even more interest was that the interlocking g−CN/CdS photoanode indicated high stabilities for the 9 h long time consecutive PEC H_2_ production test and even stabilized in ultrasonic treatment.

Apart from the CdS modification, 2D−layered transition metal sulphide (MoS_2_) has recently become an ideal co-catalyst on account of its high mobility, low cost, and narrow band gap (1.2–1.9 eV) [212,213]. Planar growth of MoS_2_ above the g−CN surface is realized due to the well-matched lattice and the similar layered structure [214,215]. The MoS_2_/S-doped g−CN heterojunction film is fabricated through CVD deposition of S-doped g−CN film on ITO glass employing thiourea and melamine as the raw materials and subsequent growth of a MoS_2_ layer by hydrothermal treatment. The obtained film showed evidently improved PEC property with the anodic photocurrent density of 1.2 × 10^−4^ A/cm^2^ at an applied potential of +0.5 V (vs. Ag/AgCl), which is approximately twice the S-doped g−CN film [216]. In addition to metal sulfide, metal phosphide semiconductors with inexpensive and earth-abundant elements have also been used to prepare g−CN-based photoelectrode [217]. Recently, Li and co-workers [206] reported the merging of gradient concentration of cobalt disulfide (CoS_2_) on PCN films by one-pot synthesis, resulting in remarkably promoted PEC performances (Figure 21e,f).

#### 6.1.3. Complex Compound/g−CN Hybrid Heterostructures Films

As we know, a complex compound consists of at least three elements. Generally, the incorporation of a complex compound into g−CN is deemed to be a promising technique for promoting photocatalytic activity, especially for the PEC, because of the efficient carrier separation and transfer [218,219,220]. For example, BiVO_4_ is an n-type semiconductor with a narrow band gap (2.4 eV), nontoxicity, low cost, and being more active in visible light [221,222,223], which is an available candidate for the development of g−CN-based heterojunction film.

Wang et al. [224] prepared nanostructured g−CN/BiVO_4_ composite films, employing facile electrospinning with a post-annealing technique. The exfoliated g−CN nanosheets prevented the agglomeration of BiVO_4_, forming heterojunctions with BiVO_4_ as well as profiting the formation of nanostructures. The obtained BiVO_4_/g−CN composite film displayed a photocurrent density of 0.44 mA cm^−2^ at 0.56 V, which was higher than that of the pristine g−CN and BiVO_4_ photoanodes. Inspired by this, Feng et al. [225] synthesized ultrathin g−CN nanosheets (g−CN−NS) and coupled them with nanoporous BiVO_4_ photoanodes via a facile ethylene glycol dispersion and impregnation. The photocurrent density of the ultrathin BiVO_4_/g−CN−NS photoanode was up to 3.12 mA/cm^2^ at 1.23 V (vs. RHE), which was approximately 7−fold and 4−fold higher than those of BiVO_4_ and BiVO_4_/bulk g−CN, respectively. The improved PEC performance was ascribed to the fact that the g−CN−NS serves as an efficient cocatalyst due to its enlarged band-gap (3.03 eV). Moreover, the intimate contact of ultrathin g−CN−NS and BiVO_4_ in core shell structure facilitated the migration and separation of the charge and restrained the interfacial charge recombination. This work accentuates the importance of geometry engineering of heterojunction film, which can be outspread to other ternary oxide semiconductor such as LaTiO_3_.

**Figure 22:**
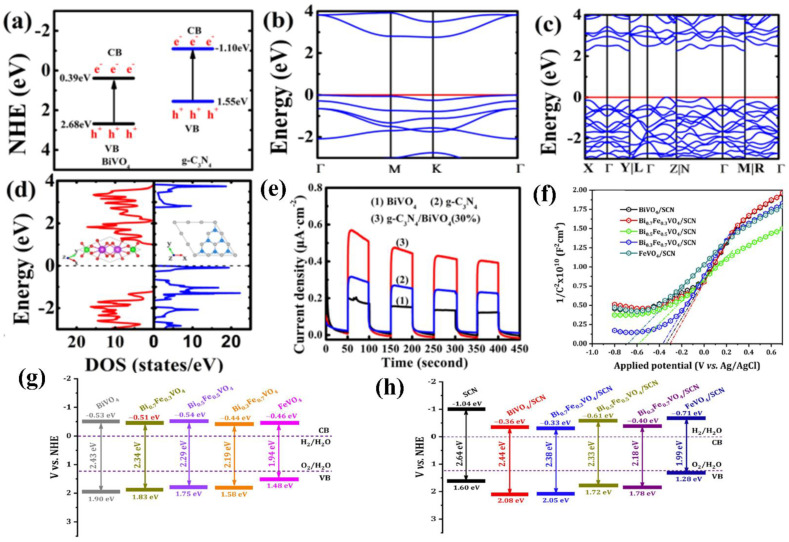
(**a**) Schematic diagram of the band alignment. (**b**) The band structure of calculated g−CN. (**c**) The band structure of calculated BiVO_4_. (**d**) Densities of sates g−CN and BiVO_4_. (**e**) Transient photocurrent response. Reprinted with permission from ref. [226] Copyright 2019 American Institute of Physics. (**f**) The electrochemical Mott-Schottky plots and (**g**,**h**) their corresponding energy level band edge diagram. Reprinted with permission from ref. [228] Copyright 2022 Elsevier.

Nevertheless, less emphasis is placed on comprehending the interface interaction and charge migration of g−CN/BiVO_4_ heterojunction. Jiang et al. [226] obtained g−CN/BiVO_4_ heterojunction photoelectrode through modified sol-gel tactics via varying the weight ratio of g−CN. The obtained g−CN/BiVO_4_ (30%) sample exhibited improved photocurrent density compared to pure BiVO_4_. Such augment can be ascribed to the effective charge separation and fast interfacial charge transfer of the type-II hetero-interface. The corresponding electronic structures and charge transfer properties were also discussed based on systematic experimental studies and density functional theory (DFT) calculations (Figure 22a–e).

By combining the elemental metal doping and hybridization with other semiconductors, Zeng et al. [227] coated the Mo-doped nanoporous BiVO_4_ surface uniformly with ultrathin g−CN through a facile electrostatic self-assembly method with improved the PEC capability. By mixing cation precursors (Bi and Fe) in different stoichiometric proportions, Yang et al. [228] performed the mixed-phase Bi_x_Fe_1–x_VO_4_ heterostructures through a one-pot hydrothermal synthesis. In addition, the impregnated Bi_x_Fe_1–x_VO_4_ on the sulfur-doped g−CN (SCN) enhances the charge separation on account of the formation of heterojunction and attains a wide range of solar absorption due to the decrease of band gap (Figure 22f–h).

**Figure 23:**
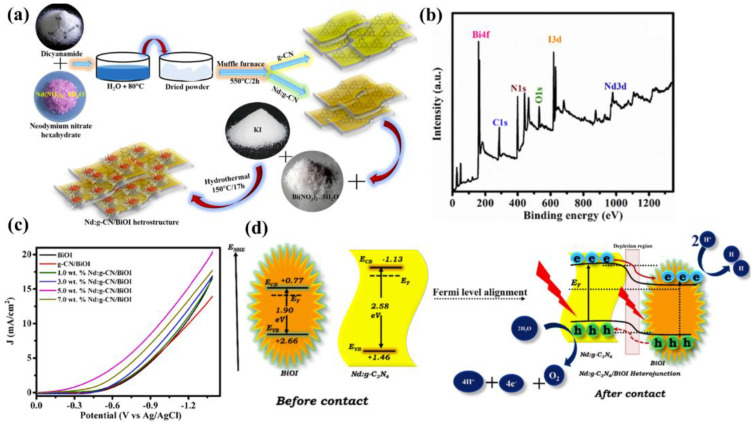
(**a**) Schematic diagram (**b**) wide scan spectrum of XPS spectra of Nd-doped g−CN/BiOI (**c**) photocurrent density versus potential curves (LSV) under light conditions. (**d**) Schematic diagram of PEC mechanism. Reprinted with permission from ref. [230] Copyright 2021 Elsevier.

A typical ABO_3_ perovskite oxide semiconductor such as LaTiO_3_ (band gap of 3.51 eV) is also well matched with g−CN to form g−CN/LaTiO_3_ heterostructure with improves the PEC activity [229]. Xing et al. [230] prepared neodymium (Nd)−doped g−CN−bismuth oxyiodide (BiOI) (Nd-doped g−CN/BiOI) through a two-step thermal poly-condensation and hydrothermal method (Figure 23a). The incorporation of Nd into the g−CN lattice decreases the band gap and also absorbs visible light, culminating in higher PEC properties (15.50 mA/cm^2^). Furthermore, the enhancement could be because of the formation of heterojunction (Figure 23b–d).

Many other complex compounds incorporate with g−CN materials have also been investigated recently, for example, g−CN/NaNbO_3_−NF [231], g−CN/CuInS_2_ [232], CdMoO_4_/g−CN [233], PbTiO_3_/g−CN [234], and all of them showed enhanced PEC activity compared to pure g−CN. Although the above materials can be used to fabricate compounds with g−CN, there still are some disadvantages: TiO_2_ can not absorb the visible light effectively due to the large band gap. Therefore, most researchers use a modified TiO_2_ to composite with g−CN, accelerating charge separation and charge transport as well as enhancing the visible light absorption of the composite material. As a sulfide photo-catalyst, the band gap of CdS is well matched with g−CN, but the photo-corrosivity and toxicity of CdS still need to be improved. Finally, the morphology of g−CN also has a great influence on PEC performance. Compared with other morphologies of g−CN, 0D semiconductor is generally more conducive to broaden the band structure, enhance the redox capacity and open circuit voltage.

### 6.2. G−CN-based Z-Scheme Heterojunction Composite Films

Although enhancive separation efficiency can be obtained based on the Type II alignment, the main drawback is that the redox ability of photo-generated charges is minimized upon the charge migration process [3,159]. Therefore, enhancing the reduction/oxidation ability is a challenge to be accomplished. Recently, the electrons migration mechanism in the Z-scheme heterojunction has been demonstrated to surmount this shortfall. By maintaining the reductive electrons in the higher CB edge and oxidative holes in the lower VB edge, it can not only enhance the charge separation efficiency but also realize a remarkably high redox ability of photo-generated charges [235,236,237,238]. The energy band structure of the Z-scheme heterojunction is similar to that of the traditional II-scheme heterojunction system. At present, the electron-hole transfer mechanism in the Z-scheme heterojunction is still controversial, and usually needs to be differentiated and verified by combining experimental and theoretical simulation. Free radical types were detected by electron paramagnetic resonance (EPR) and fluorescence spectra (PL), and the mechanism of photo-generated carrier transfer was predicted by free radical capture detection. Pan’s group [239] established synthetic tactics for the construction of g−CN/TiO_2_ heterojunction, in which g−CN was deposited on the surface of Corn-shaped TiO_2_ nanofilms via an in-site microwave-heating technique. It can be revealed that the g−CN/TiO_2_ heterojunction obviously has a higher photocurrent density compared with pure TiO_2_. The improved properties were primarily attributed to the Z-scheme heterojunction interfaces governed by the full-matched energy level between g−CN and TiO_2_. In addition, the as-designed Corn-shaped TiO_2_ nanofilms also provided a special nanostructure that may benefit from light scattering and deposition of g−CN.

Vacancy defects are reported to play a critical role in the modification of the electronic structure. Inspired by this, Xiao et al. [240] fabricated a 0D/1D g−CN nanoparticles/TiO_2_ nanotube arrays hybrid nanostructure containing an interfacial oxygen vacancy layer via anodic oxidation, NaBH_4_ reduction, and CVD methods. The prepared 0D/1D g−CN/OV−TiO_2_ possessed an optimal photocurrent density (0.72 mA/cm^2^), which was 8−fold higher than that of g−CN/TiO_2_ material (without interfacial oxygen vacancy layer). The obviously enhanced activity was accredited to the construction of Z-scheme heterostructure with an interfacial oxygen vacancy layer between TiO_2_ Nanotube arrays and g−CN nanoparticles. Furthermore, the presence of an interfacial oxygen vacancy layer provides sufficient charge carrier transfer to heterostructure.

**Figure 24:**
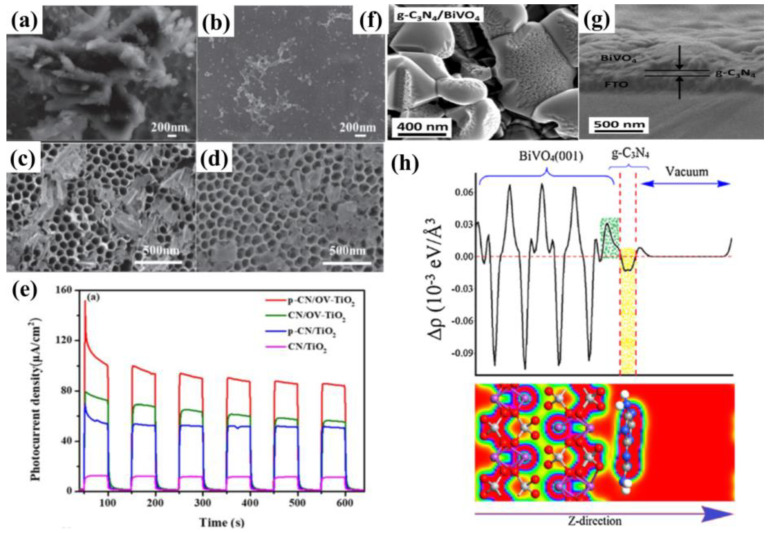
SEM images of (**a**) g−CN, (**b**) Proton-functionalized g−CN (p-CN), (**c**) g−CN/OV−TiO_2_ and (**d**) p-CN/OV−TiO_2_. (**e**) Transient photocurrent response in 0.1 M Na_2_SO_4_ at 0.6 V vs. SCE. Reprinted with permission from ref. [241] Copyright 2020 Electrochemical society. FESEM images of the top-view (**f**) and cross-section (**g**) of the BiVO_4_ electrodeposited on g−CN and (**h**) erence (Δρ) along Z-direction for g−CN/BiVO_4_. Reprinted with permission from ref. [35] Copyright 2018 Elsevier.

In another similar work by the same research group [241], the proton-functionalized g−CN (p-CN) nanosheet was deposited on the TiO_2_ nanotube array containing oxygen vacancies (OV−TiO_2_). Additionally, the protonation pretreatment was undertaken by ultrasonic bulk g−CN in the presence of hydrochloric acid, realizing larger contact areas and a close-knit contact interface between two semiconductors. The optimal photocurrent density of the obtained p-CN/OV−TiO_2_ Z-scheme heterostructure reached 96 μA cm^−2^, which was approximately 1.4 times that of CN/OV−TiO_2_ samples (Figure 24a–e). Safaei et al. [35] reported Z-scheme g−CN/BiVO_4_ photoelectrode prepared through electrodeposition of BiVO_4_ and then spin-coating on g−CN thin films surface. The optimal photocurrent of the obtained g−CN/BiVO_4_ reached 0.42 mA cm^−2^ at 1.23 V vs. RHE. For comparison, a TiO_2_/BiVO_4_ photoelectrode was also devised. The acquired photocurrent density of g−CN/BiVO_4_ is higher than that of TiO_2_/BiVO_4_, demonstrating the superiority of g−CN to TiO_2_. The enhancement was mainly because of the formation of the Vander Waals type interaction, resulting in effective charge separation at the g−CN/BiVO_4_ interface (Figure 24f–h). Furthermore, the presence of hydrogen bonds in g−CN induced the production of oxygen vacancy sites, culminating in its higher PEC properties.

**Figure 25:**
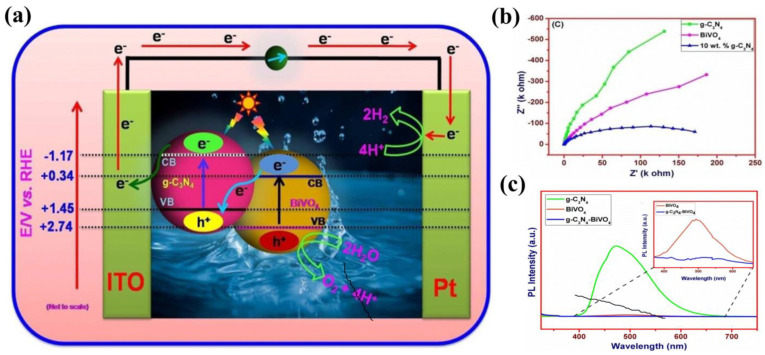
(**a**) A schematic illustration of g−CN/BiVO_4_ for overall water splitting based on direct Z-scheme heterojunction. (**b**) Photoluminescence spectra with the excitation wavelength of 280 nm (the inset shows the expanded PL spectra). (**c**) Nyquist plots. Reprinted with permission from ref. [242] Copyright 2019 Wiley-Blackwell.

In another similar study, Murugan et al. [242] designed a novel graphitic carbon nitride-bismuth vanadate (g−CN−BiVO_4_) heterojunction with different wt. % of g−CN by a simple hydrothermal approach. It can be seen from Figure 25a that the CB offset (1.51 eV) is higher than the gap between the VB of g−CN and the CB of BiVO_4_ (1.11 eV). This structure provides a strong driving force for the photo-generated electrons to transfer from the CB of BiVO_4_ to the VB of g−CN easily, leaving the photo-generated electrons and holes in the CB of g−CN and the VB of BiVO_4_, respectively. Then, the heterojunction restricts the electron–hole pair recombination, which was testified by EIS and PL spectra (Figure 25b,c). This manifests the construction of mediator-free direct Z-scheme heterojunction between g−CN and BiVO_4_. The photoanode with 10 wt. % of g−CN resulted in a photocurrent of 2.02 μA cm^−2^ at 1.23 V vs. RHE, which was higher than that of the pure BiVO_4_ and g−CN, respectively.

**Figure 26:**
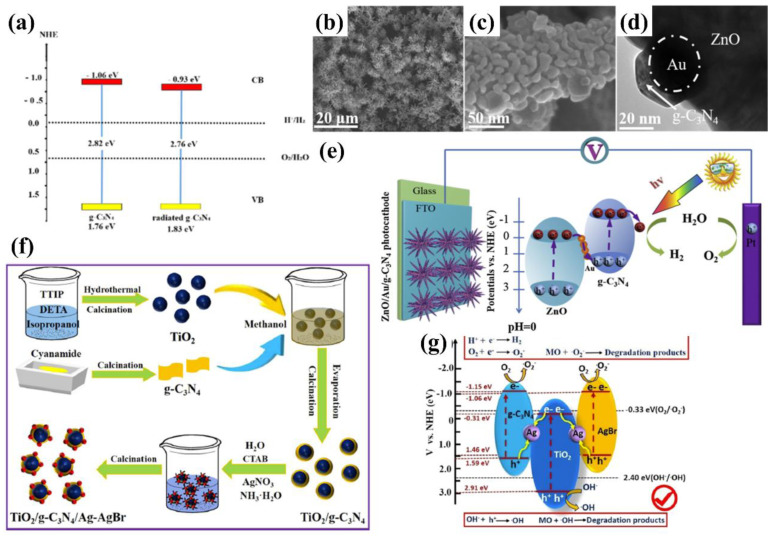
(**a**) Schematic representation of the band structures of g−CN and γ radiated g−CN. Reprinted with permission from ref. [245] Copyright 2019 American Chemical Society. SEM images of the low-magnification (**b**) and high-magnification (**c**) of the 3D urchin-like ZnO/Au/g−CN. (**d**) TEM image of 3D urchin-like ZnO/Au/g−CN. (**e**) Schematic illustration of the PEC reaction mechanism of the 3D urchin-like ZnO/Au/g−CN photocathode. Reprinted with permission from ref. [253] Copyright 2020 Elsevier. (**f**) The fabrication process of composite. (**g**) Possible photocatalytic reaction process and charge transfer of the composite. Reprinted with permission from ref. [254] Copyright 2022 Elsevier.

Tailored exposure of semiconductor materials to ionizing radiation (e.g., X-rays, α, β, γ, and neutron) can adjust the bond structure and turn the optical, electrical, and structural properties so that a higher portion of the solar spectra is captured [243]. Compared with other ionizing radiations, γ irradiation has relatively higher penetration power, no contamination and radioactive material, as well as the alteration to the chemical bonds, which is potential for solar applications [244]. A few studies have explored the positive role of γ radiation on materials. Mohamed et al. [245] reported the effect of γ irradiation on the PEC solar water splitting for the g−CN and g−CN/BiVO_4_ Z-scheme heterojunction. It has been discovered that the γ radiation alters the structural and bonding structure of g−CN, which ultimately decreases the band-gap energy from 2.82 to 2.76 eV (Figure 26a). The γ irradiated g−CN/BiVO_4_ heterojunction exhibited a high photocurrent (1.38 mA cm^−2^) at 1.23 V versus Ag/AgCl, which was higher than that of the γ irradiated g−CN or non-radiated heterojunction. Fast electron migration took place on the g−CN/BiVO_4_ heterojunction interface, which resulted from the effects of γ radiation.

NiTiO_3_, a typical ABO_3_ perovskite oxide, presents a narrow band gap (Eg = 2.2 eV), high stability in oxidizing environments, and a large absorption coefficient [246,247,248]. Moreover, NiTiO_3_ possesses appropriate band edges (E_CB_ = 0.21 eV, E_VB_ = 2.43 eV), which match well with g−CN (E_CB_ = −1.2 eV, E_VB_ = 1.5 eV) to form a direct Z-scheme heterojunction system [31,249,250]. Huang et al. [251] synthesized NiTiO_3_/g−CN under ultrasonication and subsequent calcination treatment at 400 °C for 2 h. Benefiting from a unique nanostructure and g−CN-based direct Z-scheme charge transfer mechanism, such NiTiO_3_/g−CN composite films exhibit enhanced PEC performance (0.25 mA cm^−2^) compared with the pure NiTiO_3_ and g−CN. This study provides meaningful guidance for the research of inorganic perovskite materials for water splitting, and it is essential to further inquiries into this type of photoelectrodes.

Early studies also demonstrated that the solid-state−mediators (Ag, Au, and Cu) incorporated into the interface of two components in the Z-scheme structure act as electron mediators to improve electron migration efficiency. For example, Xiao and co-workers [252] fabricated the ternary photoanode via loading g−CN on the surface of ZnO nanowire (ZnONWs) to form a junction to promote the electron tunneling effect. Whereafter, platinum nanoclusters are further incorporated at the g−CN/ZnONWs interface (g−CN/Pt/ZnO) or surface (Pt/g−CN/ZnO) by photo-depositing tactics. The photocurrent density of the Pt/g−CN/ZnO sample (120 μA cm^−2^ at 0.5 V vs. Ag/AgCl) was higher than that of g−CN/ZnO due to the cocatalyst promotion. In addition, the Pt/g−CN/ZnO photoanode showed a much lower photocurrent response than that of g−CN/Pt/ZnO, manifesting the charge carrier transportation and separation by the solid-state−mediators. Inspired by this, the 3D urchin-like ZnO/Au/g−CN Z-scheme heterostructure was prepared and applied as an excellent photocathode for PEC water splitting by Wen et al. [253]. Under simulated sunlight irradiation, both ZnO and g−CN were excited to generate electron–hole pairs. Due to the high work function (−5.30 eV) of Au nanoparticles (Au NPs), the photo-generated electrons on the CB of ZnO were quickly injected into that of Au NPs, followed by further migration into the VB of g−CN to quench the holes. The sandwiched Au NPs acted as electron transfer mediators to enhance vectorial electron migration in direct Z-scheme ZnO/g−CN heterojunction (Figure 26b–e). This work provides a brand-new insight into the application of the Z-scheme photosystem-conductor−photosystem (PS-C−PS) system for PEC water splitting, in which the high redox capacity and rapid electron transfer channel are retained. Constructing multi-heterojunction photocatalysts with multi-sources for carrier generation is one of the effective methods for enhancing the PEC activity and MO degradation. Zhao et al. [254] designed a novel TiO_2_/g−CN/Ag-AgBr (TCNAAB) multi-heterojunction by depositing Ag-AgBr nanoparticles onto 3D spherical TiO_2_/g−CN (Figure 26f–g).

The key discrepancy between Z-scheme and type II heterojunction is the electronically regulated medium for photo-carriers transfer. Compared with the traditional type II heterojunction system, the Z-scheme heterojunction maintains a strong redox capacity, achieves a wide spectral response, and possesses high stability. The main advantages of Z-scheme heterojunction are presented as follows: Firstly, there is no intermediate, it is low cost, there are fewer components, and it is easy to prepare. Secondly, the contact between the two kinds of semiconductors forms a strong interaction, which can decrease the carrier transfer resistance and improve the PEC activity. Finally, in addition to noble metal, cheaper carbon-based materials can also be used as charge modulators. By optimizing the electronic medium, electronic interface resistance, carrier transfer efficiency, and PEC stability can be enhanced. In particular, graphene and its derivatives stand out in the Z-scheme heterojunction system due to their excellent electrical conductivity and electron transport in recent years. However, Z-scheme g−CN-based heterojunction photoelectrodes are still in their elementary stage of development, and they are beneficial for future experimentation.

### 6.3. Design and Construction of Multicomponent Heterojunctions

As mentioned previously, the coupling of g−CN with appropriate semiconductors enables an efficient amelioration in the separation and migration of charge carriers via a heterojunction-transfer mechanism. Therefore, ternary g−CN-based heterojunction films will possess more meaningful applications owing to their synergistic effects.

**Figure 27:**
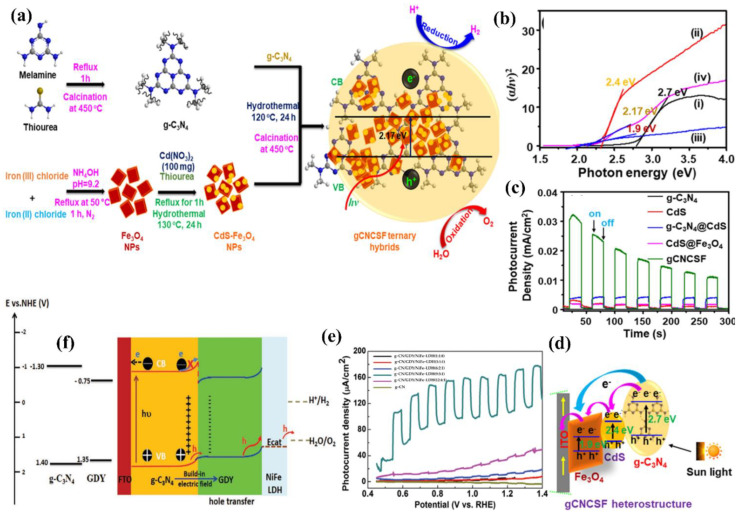
(**a**) Schematic diagram for the synthesis of g−CN@CdS–Fe_3_O_4_. (**b**) The Tauc plots. (**c**) Photocurrent response in 0.5M Na_2_SO_4_. (**d**) Schematic diagram of working mechanism at working electrode of the g−CN@CdS–Fe_3_O_4_ ternary hybrid nanocomposite. Reprinted with permission from ref. [255] Copyright 2020 Elsevier. (**e**) I-V curves for the different mass ratios of g−CN/GDY/NiFe-LDH (**a**) 1:1:8, (**b**) 3:1:1, (**c**) 6:2:1, (**d**) 9:3:1, and (**e**) 12:4:1, (**f**) g−CN). (**f**) A schematic illustration of band configuration for PEC water oxidation. Reprinted with permission from ref. [256] Copyright 2020 John Wiley and Sons Ltd.

For example, Reddy et al. [255] fabricated a g−CN−decorated CdS-nanoparticle−doped Fe_3_O_4_ nanocube (g−CN/CdS–Fe_3_O_4_) hybrid material electrode driven by a two-stage hydrothermal method (Figure 27a). Due to the well-matched electronic potentials of Fe_3_O_4_ nanocube, CdS NPs, and g−CN, the e− in the CB of g−CN were quickly migrated to that of CdS NPs, followed by further transfer into the CB of Fe_3_O_4_ nanocube to increase the charge carriers lifetime. Next, the accumulated e− in the Fe_3_O_4_ nanocube can directly transfer into the ITO-coated glass. Meanwhile, the holes on the VB of the Fe_3_O_4_ nanocube occur as a result of water oxidation. Evidently, the highest PEC efficiency in terms of linear sweep voltammetry (LSV)and photocurrent response (~0.0235 mA/cm^2^) was achieved using the g−CN/CdS−Fe_3_O_4_ electrode as the small band-gap (2.17 eV) led to effective interfacial charge transfer, which was higher than those of individual single and binary catalysts under visible-light irradiation (Figure 27b–d). In another work, g−CN/NiFe-layered double hydroxide (LDH) was modified by graphdiyne (GDY) to form a ternary hierarchical mesoporous g−CN/GDY/NiFe-LDH heterostructure [256]. Owing to the high charge mobility, unique 2D structure with sp and sp^2^−hybridized carbon atoms, and a band-gap of ~2.1 eV, the GDY can not only extend the light absorption range but also form a build-in electric field to extract and transfer the holes from g−CN to NiFe-LDH. The optimum photocurrent density for the photocathode was as high as 178.66 µA cm^−2^ at 1.4 V (Figure 27e,f). Bi_2_O_2_CO_3_/NiFe-LDH/g−CN [257] and CuTi-LDH/g−CN [258] showed enhanced PEC activity compared to pure g−CN.

**Figure 28:**
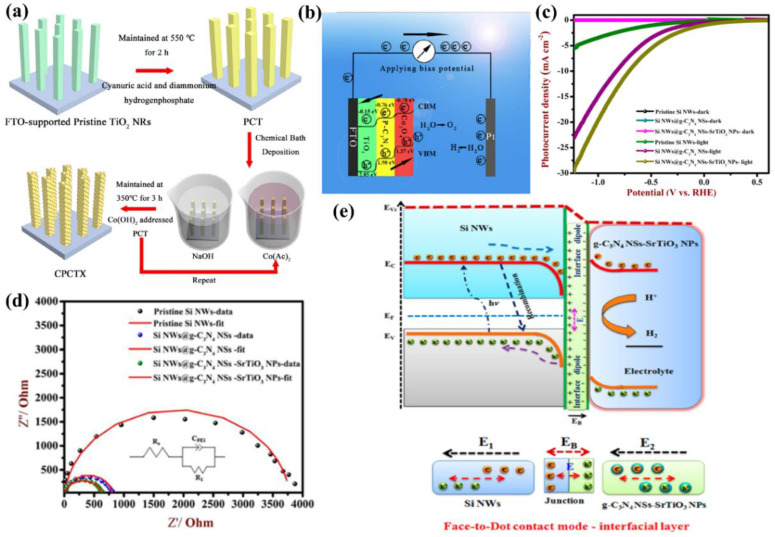
(**a**) Illustration of synthetic routine. (**b**) Schematic diagram for photogenerated charge transfer and separation in composite photoanode. Reprinted with permission from ref. [259] Copyright 2021 Elsevier. (**c**) LSV photocurrent response in 0.5 M Na_2_SO_4_ under simulated solar light irradiation. (**d**) EIS Nyquist plots. (**e**) Schematic illustration of charge transfer mechanism and photon-induced water reduction process. Reprinted with permission from ref. [262] Copyright 2019 American Chemical Society.

In addition, Zhang et al. [259] designed a 0−D Co_3_O_4_ decorated TiO_2_@P-CN (core–shell) photoanode by combining the hydrothermal strategy and successive chemical bath deposition process to enhance PEC performance. The ternary photoanode led to the rapid separation of charge to suppress the recombination rate (Figure 28a,b). The core/shell structure of CdS/g−CN/ZnO heterojunction was successfully prepared to improve the PEC activity [260].

Bashiri et al. [261] employed a two-stage hydrothermal method followed by the chemical bath deposition technique to develop a novel tailored TiO_2_/SrTiO_3_/g−CN ternary system. The photoelectrode manifests improved visible-light absorption and increased spatial separation of charge carriers. Gopalakrishnan et al. [262] employed a spin-coating strategy and subsequent annealing treatment to synthesize silicon nanowire-based hybrid heterostructures by incorporating perovskite SrTiO_3_ nanoparticle (NPs) with g−CN nanosheets (NSs) as the interfacial layer. The hybrid heterojunction as photocathode accomplished the higher photocurrent density of 28 mA cm^−2^ at 1.23 V (vs. RHE) with ABPE 5.4% at 0.8 V versus RHE. Moreover, EIS measurements suggest that the hybrid photocathode holds a reduced interfacial charge transfer resistance with a prolonged lifetime of photon-excited electrons due to the well-aligned photocathode/electrolyte interface (Figure 28c–e).

In another work, Xu et al. [263] employed a layer-by−layer assembly method to synthesize Cu_2_O/g−CN/WS_2_ triple-layer photocathode. The g−CN not only protected the inner layer but also enhanced the light-to−electricity conversion efficiency because it can form a p-n junction with Cu_2_O, while exfoliated WS_2_ was used as an out-layer catalyst for the further amelioration of the photocathode. The optimum photocurrent density under visible-light irradiation for the photocathode was as high as −9.5 mA cm^−2^ at −0.55 V versus RHE (pH 6.0). Taking advantage of CoFeO_x_ as a cocatalyst, Bhandary and coworkers [264] employed a drop-casting method to integrate hematite dendrite/carbon nitride composite (HD–CN) on an ITO glass substrate. Subsequently, CoFeO_x_ was used to modify the HD–CN through a one-step electrodeposition process. The photocurrent density could be availably improved from approximately 27 to 600 μA/cm^2^ (nearly 22 times) via the recombination of the CoFeO_x_ layer to promote electron transport. A plausible illustration for such a remarkable increment in PEC activity can be given in terms of the construction of a p–n junction between the HD–CN and CoFeO_x_ layer. The as-prepared heterojunction can help facilitate photo-generated charge separation and inhibit the electron/hole recombination.

In order to achieve efficient PEC performance, a variety of modifications have been taken to improve g−CN-based materials, for example: adjusting morphologies, metal/non-metal element doping, copolymerization, loading cocatalyst, heterojunction, etc. Ternary g−CN heterojunction film will have a wider application prospect due to its synergistic effect. However, the reasonable construction of the ternary g−CN heterojunction system needs to be optimized from the aspects of energy level structure, electronic structure and interface structure. A performance comparison of g−CN-based heterojunction composite film photoelectrodes is shown in Table 4.Cocatalysts also play an important role in ternary heterojunction. The efficient cocatalysts should meet the following conditions: (1) extract electrons from photocatalysts and transfer them to protons (H^+^); (2) excellent PEC activity for HER. To meet the first requirement, the Fermi level of the cocatalyst must be lower than the conduction band bottom of the photocatalyst, resulting in the smooth flow of photoexcited electrons and facilitating the separation of electron–hole pairs. For the second requirement, the Gibbs free energy of hydrogen adsorption on the cocatalyst should be close to zero so that proton adsorption and rapid release of hydrogen molecules with the lowest activation energy occur at neutral thermal conditions. 

### 6.4. The Stability of the g−CN Photoelectrodes for PEC Applications

The stability of photoelectrode, namely, the PEC activity does not decrease significantly during repeated recycles, is a key factor for practical PEC applications. Time-dependent current (I-t) curves under fixed bias are commonly used to evaluate stability. For semiconductor photocatalyst with poor stability, the photocurrent decreases with the increasing illumination time, and the photocatalytic properties disappear gradually (the so-called photo-corrosion). By doping with the elemental atoms, the stability of the semiconductor photocatalyst can be improved. Bian et al. [34] grew g−CN films on FTO substrates through TVC using melamine and 2,6−diaminopyridine as precursors. The successful incorporation of sp^2^ carbon groups into the g−CN film enhances the stability of photoelectrodes. The photocurrent density of the modified g−CN film is approximately 46% of that at the initial stage after 1 h and maintains 42% in the second cycle. Huang et al. [106] successfully introduced boron-doped g−CN films using boric acid as the B source. The boron-doped g−CN film retains 90% initial photocurrent under 10 h illumination while only 7% for the pristine one. This is mainly due to the efficient extraction of photo-generated holes from the surface region, reducing the chemical attack on the C-N bond. The cocatalyst is also a fascinating strategy to enhance the stability of the semiconductor photocatalyst and improve the PEC performance. Cocatalysts act as a protection layer to catalyze the surface reactions by minimizing the activation energies and facilitating the separation of photo-induced charge. For example, the nickel incorporated sulfur-doped g−CN with bismuth oxybromide (Ni/S-g−CN/BiOBr) photoelectrode exhibits a photocurrent density of 1 ± 0.2 mA cm^−2^ under illumination for 6000 s [13]. To achieve a long-term stability, the modified technologies should be further studied, including protecting g−CN from photo−/chemical-corrosion by protective layers and using both co-catalyst and protective layers. The reduced graphene oxide (rGO) can act as an electron transfer channel for reducing the recombination of the electron–holes and a protective layer for protecting g−CN from photo−/chemical-corrosion. Therefore, the rGO can be introduced into g−CN-based photoelectrodes in order to achieve good stability.

## 7. Conclusions, Perspective, and Outlook

**Figure 29:**
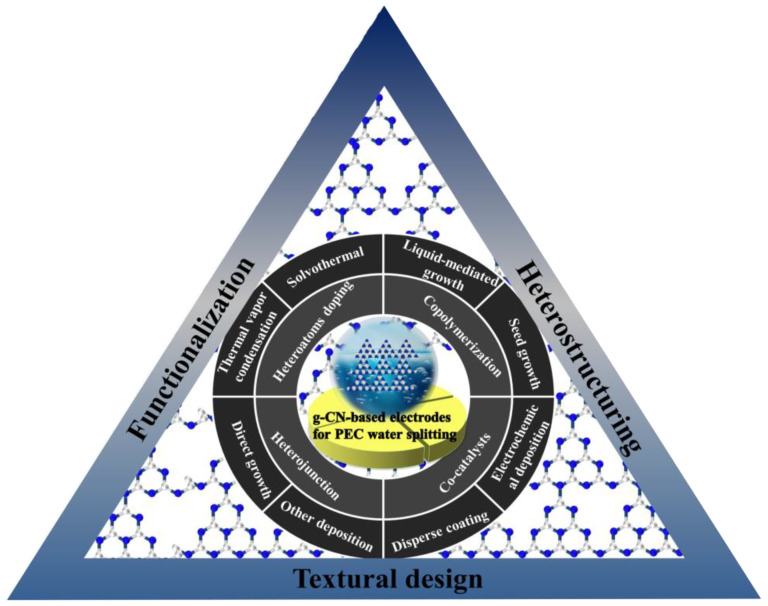
Schematic illustration of the topics involved in this review.

The low price, high physicochemical stability, and attractive electronic band structure of g−CN materials, together with the recent developments in their controlled growth on various substrates, contribute to expectations of an infusive future for g−CN materials in PEC applications. The overview of the topics involved in this review is illustrated in Figure 29. In contrast to the many groundbreaking results on g−CN powders and their demonstrated functionality in various applications to date, the research reports on g−CN materials in photoelectrochemical applications are still in their infancy. To this end, to achieve a significant advancement in this topic and to obtain efficient photoelectric devices requires resolving the following points:

1. Improvement in the deposition methods:

Currently, it is still difficult to achieve a final film that is both homogenous and with good contact with the conductive substrate while concurrently controlling morphology, electronic structure, as well as optical and PEC properties. The deposition methods should be further modified in the aspect of the starting materials design, interfaces, and reactor parameters optimization for achieving the g−CN layers in a more controllable manner. Morphology regulation is the most direct, simple and effective method to improve the electronic structure, surface characteristics, and photoelectrical properties of g−CN. The three-dimensional (3D) sphere or cage structure of g−CN has a larger specific surface area and sufficient reactive sites but has not been explored in the field of PEC water splitting. Compared with other morphologies (bulk, flake and thin film), 0D semiconductor is generally more helpful in broadening the band structure, enhancing the redox ability and open-circuit voltage.

2. Tuning the band gap of g−CN:

Decreasing the band gap of g−CN would result in increased light absorption and photocurrent density. In general, it can be acquired by optimizing the C/N ratio or the spatial organization of the N atoms or by doping with heteroatoms and halogens. The electronic structure of g−CN materials can be modified by inducing vacancy defects or tailoring monomer design.

3. Improvement of photo-excited charge separation and suppression of the recombination of the carrier:

The low electronic conductivity of the g−CN layer leads to a high photo-excited charge recombination rate and to a low diffusion length. The latter restricts the thickness of the active layer and hence limits light absorption. While increasing the thickness of the active harvesting layer results in enhanced light absorption, this also results in higher charge recombination rates. This trade-off is crucial in the PEC efficiency and is restricted by light absorption and charge extraction properties. Replacing some C=N with C=C bonds or doping with heteroatoms/metals could highly improve the conductivity of electrons. Plenty of theoretical and experimental research has proved that the formation of well-designed g−CN-based heterostructures can improve its charge separation and transfer within the electrode. Constructing multi-heterojunction photocatalysts with multi-sources for carrier generation is one of the effective methods for enhancing the PEC activity. Cocatalysts also play an important role in enhancing charge carrier transfer. Two-dimensional transition metal sulfide has a unique electronic structure and large work function, which is considered a potential cocatalyst to replace precious metals. It has the following advantages: (1) a large number of active sites located at both edge and substrate; (2) strong conductivity to promote the rapid transfer of photogenerated electrons; (3) the edge position has higher H^+^ adsorption capacity and accelerates the adsorption and desorption of hydrogen.

4. Enhancing the stability of g−CN:

In a photoanode, water oxidation on the semiconductor surface is still the bottleneck that needs to obtain more attention in future studies. The introduction at the g−CN/electrolyte interface of a co-catalyst that can both serve as the catalytic sites for oxygen evolution and act as an extract for holes from the g−CN was demonstrated to be a successful tactic. There are several factors that should be taken into account when incorporating a co-catalyst onto a g−CN layer. One problem is the nature of the contact, in which different types of co-catalysts change the transport of photo-induced charges across the g−CN/electrolyte interface. On the other hand, the electronic properties of the co-catalyst need to match the VB potentials of the CN for promoting hole transfer. To achieve a long-term stability, the modified technologies should be further studied, including protecting g−CN from photo− or chemical-corrosion by protective layers and using both a co-catalyst and a protective layer is possible.

To summarize, although there is a mass of obstacles hindering and taking advantage of carbon nitride materials in PEC devices, we believe that their excellent and unique physicochemical properties will enable us to conquer the current limitations. We believe that enhancements in the synthesis and deposition tactics, as well as a better understanding of their photoelectrochemical and charge separation and transfer processes by the study of structure-activity relationships, will result in their exploitation in many optoelectronic devices.

## Figures and Tables

**Table 1:** The main advantages and disadvantages comparison of different methods.

Synthesis Methods	Advantages	Disadvantages
Thermal vapor condensation (TVC)	(i) Facile and inexpensive. (ii) Produced uniform and dense g−CN film.	(i) Limited precursors (Other frequently reported precursors fail to form g−CN films on solid substrates). (ii) Limited thickness. (iii) The temperature of deposition is limited by the different substrates.
solvothermal	(i) Uniform deposition. (ii) Controllable thickness.	(i) Complicated preparation process. (ii) The requirement of long reaction times. (iii) Produced g−CN film with rich surface defects.
Liquid-mediated growth	(i) Very facile and cheap to perform. (ii) Strong adhesion of the g−CN film to the substrate.	(i) The temperature of deposition is limited by the different substrate and precursor evaporation. (ii) Hard to control the film thickness.
Seed growth	(i) Facile and inexpensive.	(i) Complicated preparation process. (ii) Produced g−CN film with high roughness.
Electrochemical deposition	(i) The temperature of deposition is not limited by the different substrates.	(i) Hard to produce a uniform g−CN film. (ii) Physical adhesion of g−CN to the different substrates.
Disperse coating	(i) Facile and cost-effective. (ii) Continuous, homogeneous coating formed on various substrates. (iii) Easy to control the film thickness. (iiii) The temperature of deposition is not limited by the different substrates.	(i) The film is easy to fall off. (ii) Limited scalability due to the poor solubility of g−CN powder in most solvents. (iii) Physical adhesion of g−CN to the different substrates. (iiii) Produced g−CN film with rich surface defects.
Anodic aluminum oxide membrane deposition	(i) Continuous, homogeneous coating formed on various substrates. (ii) Controllable thickness	(i) Complicated preparation process.
electrospinning	(i) Facile. (ii) Controllable thickness.	(i) Weak adhesion of the g−CN film to the substrate. (ii) The stability of the organic matrix is lower than pure g−CN.
vacuum magnetic filtered arc ion plating	(i) Facile. (ii) Controllable thickness. (iii) Prepared g−CN film with relatively high crystallinity.	(i) Expensive equipment. (ii) Plasma instability.
Radio frequency magnetron-based sputtering	(i) Facile and highly efficient. (ii) Prepared g−CN film with relatively high crystallinity. (iii) Controllable thickness.	(i) Expensive equipment. (ii) Plasma instability.

**Table 2:** Photocurrent response comparison of different methods.

Method	Precursor	Photoelectrode	Potential (V)	J (μA cm^−2^)	Electrolyte	Light Source	Ref.
TVC	melamine	g−CN	1.55 vs. RHE	120	0.1 M Na_2_SO_4_ + 0.01 M Na_2_S + 0.1 M Na_2_SO_3_	AM 1.5	[48]
TVC	dicyanamide	g−CN	1.23 vs. RHE	63	0.1 M Na_2_SO_4_	AM 1.5	[49]
SR	cyanuric chloride + melamine	g−CN	1.23 vs. RHE	3.5	0.2 M Na_2_SO_4_	Visible light (λ ≥ 420 nm)	[57]
SR	cyanuric chloride + cyanuric acid	g−CN	1.23 vs. RHE	10	0.5 M Na_2_SO_4_	AM 1.5	[58]
LMG	Melamine + Trithiocyanuric acid	g−CN@sulfur	1.23 vs. RHE	100	1.0 M NaOH	AM 1.5	[64]
LMG	2,4−diamino−6−phenyl−1,3,5−triazine + Cyanuric acid + Ni chloride salt	g−CN:nickel	1.23 vs. RHE	69	0.1 M KOH	AM 1.5	[65]
SG	Melamine	g−CN	1.23 vs. RHE	116	0.1 M KOH	AM 1.5	[66]
EPD	cyanuric acid-melamine	Carbon fiber/g−CN	0 vs. Ag/AgCl	12	1 M KOH	50 W white LED (λ > 410 nm)	[71]
ESP	Dicyandiamide + PVP	g−CN@PVP	0.5 vs. SCE	6.64	0.05 M Na_2_SO_4_	AM 1.5	[77]
AAO	cyanamide	g−CN	1.23 vs. RHE	30.2	0.1 M Na_2_SO_4_	AM 1.5	[90]
VMFAIP	Graphite targets and nitrogen gas	g−CN	1.23 vs. RHE	10.8	0.5 M Na_2_SO_4_	AM 1.5	[93]
RFM	g−CN pellet targets + Ar plasma	g−CN/TiO_2_	0.9 V vs. Ag/AgCl	290	10 mM NaOH	Visible light (l > 420 nm)	[95]

TVC: thermal vapor condensation; SR: solvothermal route; LMG: liquid-mediated growth; SG: seed growth; EPD: electrophoretic deposition; DC: disperse coating AAO: Anodic aluminum oxide membrane deposition; ESP: electrospinning; VMFAIP: vacuum magnetic filtered arc ion plating; RFM: Radio frequency magnetron based sputtering.

**Table 3:** Representative summary of the PEC activity enhancement of g−CN-based photoelectrodes.

Photoelectrode	Dopant/g−CN Precursor	Potential (V)	J (μA cm^−2^)	Electrolyte	Light Source	Ref.
C/g−CN	2,6−Diaminopyridine	1.23 vs. RHE	100	0.1 M Na_2_SO_4_ + 0.01 M Na_2_S + 0.1 M Na_2_SO_3_	AM 1.5	[53]
Ag/g−CN	AgNO_3_	1 V vs. RHE	6.40 μA/mm^2^	0.05 M Na_2_SO_4_	Visible light (l > 420 nm)	[102]
Pd/g−CN	Pd(NO_3_)_2_	1.23 vs. RHE	79.2	0.5 M Na_2_SO_4_	Visible light (l > 420 nm)	[103]
B/g−CN	H_3_BO_3_	1.23 vs. RHE	55	0.2 M Na_2_SO_4_ + 0.05 M Na_2_S	AM 1.5	[106]
P/g−CN	hexachlorocyclot-riphosphazene	1.23 vs. RHE	120	1 M NaOH	AM 1.5	[114]
S/g−CN	ammonium thiocyanate	1.23 vs. RHE	110	1 M NaOH	AM 1.5	[116]
K&I/g−CN	KI + I_2_	0.5 V versus Ag/AgCl	50.3	0.25 M Na_2_S + 0.35 M Na_2_SO_3_	Visible light (l > 420 nm)	[121]
layered-doped g−CN	H_3_BO_3_+H_3_PO_4_	1.23 vs. RHE	150 ± 10	0.1 M Na_2_SO_4_	AM 1.5	[122]
g−CN	melamine+2,6−diaminopyridine (26D)	1.23 vs. RHE	100	0.1 M Na_2_SO_4_ + 0.1 M Na_2_SO_3_ + 0.01 M Na_2_S	AM 1.5	[53]
g−CN	melamine + cyanuric chloride	1.23 vs. RHE	230	0.5 M Na_2_SO_4_	AM 1.5	[134]
g−CN	Melamine + formaldehyde	1.23 vs. RHE	228.2	0.2 M Na_2_SO_4_	AM 1.5	[135]
C_3_N_5_/TiO_2_	Melamine + hydrazine hydrate	1.23 vs. RHE	152	0.1 M Na_2_SO_4_	AM 1.5	[136]
g−CN/TiO_2_	Melamine + hydrazine hydrate	1.23 vs. RHE	100	0.1 M Na_2_SO_4_	AM 1.5	[136]
g−CN g−CN/FeOOH	thiourea urea	1.1 vs. RHE 1.1 vs. RHE	89 122	0.1 M Na_2_SO_4_ 0.1 M Na_2_SO_4_	AM 1.5 AM 1.5	[51]
g−CN/NiCo-LH	cyanuric chloride melamine	0.6 V vs. SCE	11.8	0.2 M Na_2_SO_4_	200 W xenon lamp	[51]
g−CN g−CN@CNT	melamine	0 vs. SCE 0 vs. SCE	0.5 1.8	0.5 M Na_2_SO_4_ 0.5 M Na_2_SO_4_	ZOLIX, 150 W, AAA	[91]
DPCN/NRGO/NiFe-LDH	urea+hydrogenation treatment	1.4 vs. Ag/AgCl	162.3	0.01 M Na_2_SO_4_	AM 1.5	[142]
CN-rGO	cyanuric acid melamine	1.23 vs. RHE 1.23 vs. RHE	72 660	0.1 M KOH 0.1 M KOH + 10% (*v*/*v*) TEOA	AM 1.5 AM 1.5	[145]

**Table 4:** Comparison of g−CN-based heterojunction photoelectrodes for PEC application.

Photoelectrode	Composite Type	Potential (V)	J (μA cm^−2^)	Electrolyte	Illumination	Ref
mesoporous TiO_2_/g−CN	Type II	1.23 V vs. RHE	320	0.5 M Na_2_SO_4_	AM 1.5	[167]
mesoporous TiO_2_/g−CN	Type II	0 vs. Ag/AgCl	1400	0.1 M Na_2_S	Visible light (l > 410 nm)	[168]
g−CN/TNTA	Type II	1.23 V vs. RHE	860	0.1 M Na_2_SO_4_	AM 1.5	[174]
TiO_2_/g−CN	Type II	1.23 V vs. RHE	2740	1 M NaOH	AM 1.5	[175]
P-CN/TiO_2_ NTs	Type II	0 V vs. Ag/AgCl	1980	1 M NaOH	AM 1.5	[176]
g−CN/H-S−TiO_2_ NTAs	Type II	1.23 V vs. RHE	1640	1 M KOH	AM 1.5	[178]
CNQDs/TiO_2_ NTAs	Type II	0.3 V vs. Ag/AgCl	1340	0.1 M Na_2_SO_4_	AM 1.5	[184]
Y:ZnO/g−CN	Type II	1.23 vs. RHE	400	0.5 M Na_2_SO_4_	AM 1.5	[188]
ZnONW/g−CN	Type II	1.23 vs. RHE	250	0.1 M KOH	AM 1.5	[190]
ZnO/g−CN−QDs	Type II	0.5994 V versus RHE	952	0.5 M Na_2_SO_4_	AM 1.5	[191]
g−CN/Ti−Fe_2_O_3_	Type II	1.23 vs. RHE	2550	1 M NaOH	150 mW/cm^2^	[193]
g−CN/WO_3_	Type II	+2.0 V vs. RHE	2100	0.2 M Na_2_SO_4_	AM 1.5	[194]
WO_3_/g−CN NSAs	Type II	1.23 vs. RHE	730	Sea water	AM 1.5	[195]
g−CN/WO_3_	Type II	1.23 vs. RHE	1920	0.1 M KH_2_PO_4_	AM 1.5	[196]
g−CN/Fe_2_O_3_	Type II	1.23 vs. RHE	800	1 M NaOH	150 mW/cm^2^	[197]
CdS NRs/g−CN NSs	Type II	1.23 vs. RHE	100	0.05 M Na_2_SO_4_	150 mW/cm^2^	[208]
CdS@g−CN CSNRs	Type II	0.9 vs. RHE	1160	0.35 M Na_2_SO_3_ +0.25 M Na_2_S	AM 1.5	[210]
g−CN/CdS	Type II	0.0 V bias vs. Ag/AgCl	5400	0.5 M Na_2_S +0.5 MNa_2_SO_3_	AM 1.5	[211]
MoS_2_/S-doped g−CN	Type II	0.5 vs. SCE (+0.5 V vs. Ag/AgCl)	120	0.1 M Na_2_SO_4_	AM 1.5	[216]
CoP/g−CN	Type II	+0.40 V (versus Ag/AgCl)	150	0.05 M Na_2_SO_4_	AM 1.5	[217]
g−CN/BiVO_4_	Type II	1.23 vs. RHE	440	0.5 M KH_2_PO_4_+ 1 M Na_2_SO_3_	Visible light (l > 420 nm)	[224]
BiVO_4_/g−CN−NS	Type II	1.23 vs. RHE	3120	0.1 M Na_2_SO_4_	Visible light (l > 420 nm)	[225]
g−CN/Mo: BiVO_4_	Type II	1.23 vs. RHE	3110	0.1 M KH_2_PO_4_	AM 1.5	[227]
0D/1D g−CN/OV−TiO_2_	Z-Scheme	1.23 vs. RHE	720	0.1 M Na_2_SO_4_	Visible light (l > 420 nm)	[240]
p-CN/OV–TiO_2_	Z-Scheme	0.6 V vs. SCE.	96	0.1 M Na_2_SO_4_	Visible light (l > 420 nm)	[241]
g−CN−BiVO_4_	Z-Scheme	1.23 vs. RHE	2.02	1 M KOH	AM 1.5	[242]
g−CN/BiVO_4_	Z-Scheme	1.23 vs. RHE	420	0.5 M Na_2_SO_4_	AM 1.5	[35]
g−CN@BiVO_4_	Z-Scheme	1.23 vs. RHE	1380	0.5 M Na_2_SO_4_	AM 1.5	[245]
NiTiO_3_/g−CN	Z-Scheme	1.23 vs. RHE	250	0.1 M Na_2_SO_4_	30 W visible–light LED	[251]
g−CN/Pt/ZnO	Semiconductor-conductor−semiconductor Z-scheme	0.5 V vs. Ag/AgCl	120	0.5 M Na_2_SO_4_	AM 1.5	[252]
ZnO/Au/g−CN	Semiconductor-conductor−semiconductor Z-scheme	0 V vs. RHE	−290	0.2 M Na_2_SO_4_	AM 1.5	[253]
g−CN@CdS–Fe_3_O_4_	ternary	0.2 V vs. Ag/AgCl	23	0.5 M Na_2_SO_4_	AM 1.5	[255]
g−CN/GDY/NiFe-LDH	ternary	1.4 V	178.66	0.01 M Na_2_SO_4_	AM 1.5	[256]
CdS/g−CN/ZnO	ternary	1.23 vs. RHE	3340	0.1 M Na_2_S + 0.2 M Na_2_SO_3_	Visible light (l > 420 nm)	[260]
SiNWs@g−CNNSs−SrTiO_3_ NPs	ternary	1.23 vs. RHE	−28,000	0.5 MNa_2_SO_4_	AM 1.5	[262]
Cu_2_O/g−CN/WS_2_	ternary	−0.55 vs. RHE	−9500	1 M Na_2_SO_4_	Visible light (l > 420 nm)	[263]
CoFeO_x_/HD/g−CN	ternary	1.23 vs. RHE	600	1MNaOH	Visible light (l > 420 nm)	[264]

## Data Availability

The data presented in this study are available on request from the corresponding author.
